# Advancing physical literacy measurement in early childhood: psychometric properties of a novel assessment and profiling method and its relationship with physical activity

**DOI:** 10.3389/fspor.2026.1773645

**Published:** 2026-03-19

**Authors:** Natalie Weir, Richard Tyler, Andy Pringle, Clare M. P. Roscoe

**Affiliations:** 1 Clinical Exercise Rehabilitation Research Centre, School of Sport and Exercise Science, University of Derby, Derby, United Kingdom; 2 Sport, Physical Activity, Health, and Wellbeing Research Group, International Centre for Applied Research with Children, Young People, Pregnant Women and Families (iCARE), Department of Sport and Physical Activity, Edge Hill University, Ormskirk, United Kingdom

**Keywords:** assessment, early years, education, physical activity, physical literacy, pre-school, psychometrics

## Abstract

**Introduction:**

Physical literacy provides a compelling framework for understanding young children's skills, attitudes, and behaviours in relation to movement and physical activity. Assessment represents a crucial step for establishing conceptual and practical relevance. However, there is limited evidence to support how this multidimensional construct can be meaningfully assessed in early childhood, constraining both research advancement and applied intervention.

**Methods:**

This mixed-methods study primarily aims to establish evidence for the Physical Literacy Early Years (PLEY) Wheel, a novel assessment approach assessing physical, social, cognitive, and affective skills and behaviours in children aged 3–5 years. 234 children (74% White British; age 3.84 ± 0.70 years; 51% girls, IMD decile 3.70 ± 3.0) and 17 educators (88% female) from 15 early education settings participated across a multi-phase study design. Secondary analyses examine associations with key variables, including device-based measured physical activity, and explore profile-based representations to support interpretation.

**Results:**

The PLEY Wheel provides a valid, reliable, and practical approach to benchmarking and profiling physical literacy in early childhood. The results showed that physical literacy increased with age, with girls scoring higher in cognitive and social domains, and children with special education needs or disabilities demonstrating lower physical literacy and physical activity. Physical activity varied widely within and between early education settings and was associated with physical literacy. Five profiles were identified, based on domain strengths and weaknesses, that were sensitive to age, sex, and socio-economic context, but did not strongly differentiate physical activity levels.

**Discussion:**

These findings advance the understanding of physical activity and physical literacy in 3–5-year-old children and highlight the potential of a child-centred, data-led approach. Establishing a valid and reliable assessment tool for early childhood provides a methodological foundation for examining how physical literacy manifests, relates to physical activity, and can be meaningfully interpreted to support early intervention.

## Introduction

The rising prevalence of sedentary behaviour and negative attitudes towards physical activity (PA) (1) pose significant challenges to the development, health, and wellbeing of young children. In early childhood, PA typically occurs through short bursts of active play ranging from light-intensity physical activity (LPA) to moderate-to-vigorous intensity physical activity (MVPA). Guidelines in the United Kingdom recommend that children aged 3–5 years accumulate at least 180 min of daily PA, including 60 min of MVPA, i.e., heart pumping, faster breathing, and energetic play (2). Sufficient PA during this period of rapid neurological and physical development contributes to motor competence and movement confidence (3) as well as cognitive functioning (4). This includes attention, working memory, academic performance (5), and self-regulation (6). In the context of rising sensory processing challenges, cognitive functioning and communication difficulties, and pressure for children to reach a “good level of development” by 5 years of age (7), the consequences of inactivity are increasingly urgent. This highlights the need for meaningful movement experiences in early childhood.

Physical literacy (PL) provides a compelling concept for understanding and supporting young children's meaningful engagement with movement and PA (8). The International Physical Literacy Association defines PL as the motivation, confidence, physical competence, knowledge, and understanding to value and take responsibility for engagement in physical activities for life (9). In England, the PL consensus statement describes PL as the extent to which we have a meaningful, positive relationship with movement and physical activity throughout life. Specifically, engagement in PA is shaped by how we move, connect, think, and feel through movement experiences (10). Emerging evidence links higher PL with positive wellbeing outcomes (11), increased PA and lower sedentary time (12), and stronger motor competence (13, 14). Indeed, PL is often incorrectly defined or assessed as the ability to perform movement skills, focusing on the physical domain, despite the multidimensional and personalised nature of PL (15). In addition, research has yet to establish the essential capabilities and competencies that characterise early childhood PL (16).

Assessment represents a crucial next step for establishing conceptual and practical relevance of PL, informing intervention (17, 18), and addressing the skills and knowledge gap that exists within an early education context (19, 20). However, with over 20 competing definitions of PL identified (21), challenges exist in assessing PL as a holistic construct (22, 23). Tools developed for an early education context often focus on educator knowledge, pedagogy, and practice (24, 25). Existing methods to benchmark children's PL differ conceptually (26), disproportionately focus on the physical domain of PL, or omit key domains completely, such as the social domain (27). Adaptations of broader PL tools often require lengthy, specialist administration (28), lack developmental suitability for younger children (29), or inadequately report psychometric validation (30). Of the two child-level, product-focused assessment tools identified within the literature as used within the preschool population, the Preschool Physical Literacy Assessment Tool (Pre-PLAy) (31) does not include all domain areas and disproportionately measures physical domain components. The Physical Literacy in Children Questionnaire (PL-C Quest) (32) is a pictorial-based assessment of children's perceived PL widely used and validated for children aged 4–12 years (27). However, the time burden for children to evaluate themselves across 30 items (40% physical) and young children's limited capacity for reliable self-report (33) constrain feasibility and applicability for use with younger children. In addition, linear PL assessment scoring methodologies fail to recognise that strengths and weaknesses across domains may combine in different ways (34, 35). With 83% of children aged 3 years and 91% of children aged 4 years receiving formal childcare in England in 2024 (36), 3–5-year-old children are likely to spend a substantial proportion of their time in early childhood education and care (ECEC), under the supervision of educators. Educators have been deemed best placed to administer PL assessment in an educational context (37). PA levels have also been shown to peak around the age children start school and decline thereafter (38).

**Figure 1:**
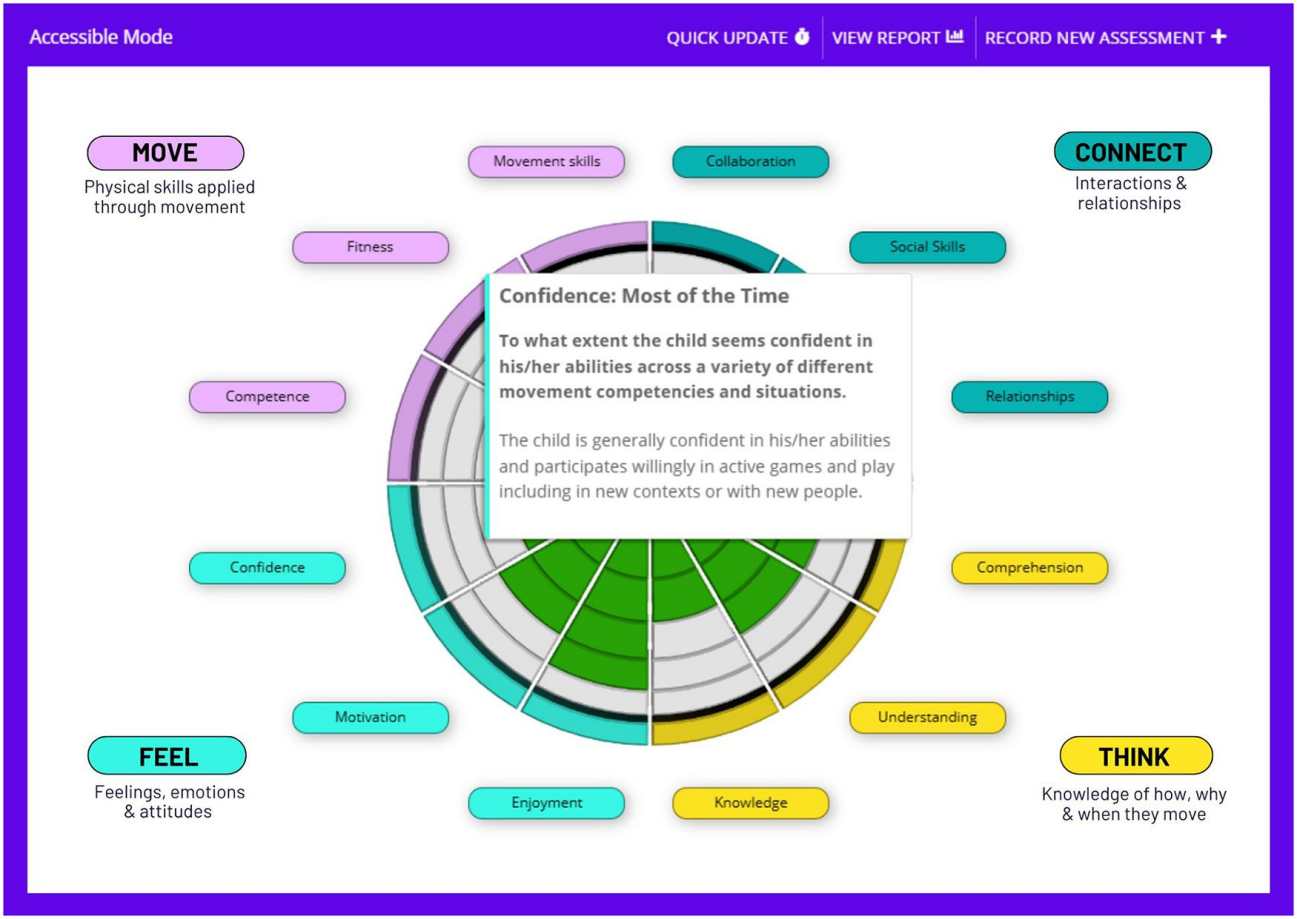
Visualisation of the Physical Literacy Early Years (PLEY) wheel tool.

Improving the understanding of how PL manifests in 3–5-year-olds, alongside a stronger objective measurement of current PA levels at this age, is important for informing future policy and intervention development. Yet, to the authors' knowledge, there is no tool designed specifically for early childhood that is psychometrically robust, applies equal weighting across physical, social, cognitive, and affective domains, and meets the need for practical population-level and intervention study use (39). Therefore, research is needed to establish a reliable and valid method for benchmarking PL in 3–5-year-olds that addresses the lack of social domain integration and over-reliance on motor skill–based assessments and enables exploration of its relationship with PA. This study presents and examines the psychometric properties of the Physical Literacy Early Years (PLEY) Wheel tool (see Figure 1), guided by the Consensus-based Standards for the Selection of Health Measurement Instruments (COSMIN) checklist (40). The study aims are 3-fold: (1) to establish evidence for the PLEY Wheel's construct validity, test–retest reliability, and internal consistency in a sample of 3–5-year-old children in England; (2) to examine its associations with key variables, including objectively measured physical activity; and (3) to generate profile-based representations of PL to support understanding and application in early childhood contexts.

## Methods

A convenience sampling approach was used to recruit ECEC settings to participate in this study. This was facilitated through the researcher's relationships with Early Years (EY) networks and partner organisations. The aim was to opportunistically recruit ECEC educators and a diverse sample of 3–5-year-old children from preschools, private nurseries, or primary school reception classes. Socio-economic status was quantified using the UK Index of Multiple Deprivation (IMD), an area-level measure of deprivation based on income, employment, health, education, crime, housing, and living environment. IMD decile 1 indicates the greatest area-level deprivation and decile 10 the least deprived. Following setting/gatekeeper consent, parental consent, and child assent (parental consent rate 60%), 234 children (74% White British; age 3.84 ± 0.70 years; 51% girls, IMD decile 3.70 ± 3.0) and 17 educators (88% female) from 15 settings (IMD decile range 1–10) located in Derbyshire, Manchester, and Lancashire in England, UK, participated in this study. Ethical approval was provided by the College of Science and Engineering Research Ethics Committee, University of Derby (ETH2324-4649).

A multi-phase design was used for this study. Phase 1 (June to December 2024) involved a cross-sectional assessment of PA and PL in 203 children [76% White British, age 3.87 ± 0.72 years, 52% girls, body mass index (BMI) Centile 57.45 ± 27.27, IMD decile 3.60 ± 2.92] from 10 Derbyshire ECEC settings. Located in both urban and rural locations, they included four preschools on primary school sites, two private nurseries, and four primary school reception classes. PA was measured using triaxial accelerometers worn whilst in the education environment for 5 consecutive days. The PLEY Wheel tool was completed by educators (*n* = 12) to assess children's PL. IMD and demographic data (such as age and ethnicity), educational need indicators, including English as an additional language (EAL), pupil premium eligibility (PPI), and special educational needs or disabilities (SEND) were provided by each setting prior to PA and PL data collection. Anthropometric data were measured by using a portable stadiometer to the nearest 0.5 cm for height and Tanita DC-240 portable weighing scales (Tanita Inc., Tokyo, Japan) to the nearest 0.1 kg for body mass. BMI was calculated and categorised according to standardised, age-appropriate thresholds (41). Finally, contextual, pedagogical, and process data were collected via a debrief survey.

Phase 2 (May to July 2025) comprised a series of psychometric validation and reliability activities for the PLEY Wheel tool, including test–retest reliability, intra-rater reliability, face and content validity, and additional response process data. Educators (*n* = 5) from five ECEC settings completed the PLEY Wheel on two occasions for a minimum of six children, separated by a 14-day interval. This test–retest interval aligns with established practice in the validation of PL and related behavioural assessment tools in early childhood (42) and added 31 children (age 3.81 ± 0.72 years, 52% boys) to the overall study sample for validity and reliability analyses. A survey to investigate face and content validity (Supplementary Material 1) captured wider views on the clarity, appropriateness, and practicality of the PLEY Wheel tool. This included both quantitative and qualitative components. Quantitative items used five-point Likert-scale questions to assess agreement with specific statements, for example: The profiles shown are easy to understand and interpret, 1 = “strongly disagree,” 5 = “strongly agree.” Open-ended questions (qualitative items) invited participants to elaborate on perceived strengths, areas for improvement, and practical considerations for implementation. The survey was designed for accessibility and relevance to EY educators, researchers, and stakeholders and included a 2-min video providing an overview on how to complete the PLEY Wheel and accompanying downloadable documents. Stakeholder agreement was defined as ≥70% of respondents selecting the top two Likert-scale options (e.g., “agree” or “strongly agree”), indicating acceptable consensus. The survey was disseminated through the researcher's professional networks, via targeted email invitations and social media channels. These included the International Physical Literacy Association (IPLA), National Early Years Active Start Partnership (NEYASP), Active Partnerships, and Sport England PL consensus statement stakeholders. Participants (*n* = 90, 61% female) included academics (*n* = 40), educators (*n* = 38), and strategic/policymakers/other (*n* = 31), with a minimum of 2 years of experience in PL, PA, physical development, or EY education and development.

### Accelerometry

Children's PA was measured using Actigraph GT3X + accelerometers (Actigraph, Florida, USA). A 100-Hz sampling rate was adopted (43) to best capture the sporadic activity of 3–5-year-old children (44). The non-dominant wrist was chosen for wear location because of expected higher compliance in this age group (45). Activity levels were classified as sedentary behaviour (SB), LPA, and moderate-to-vigorous physical activity (MVPA) according to vector magnitude (VM) cut points validated for preschoolers (46) as follows: SB (VM = 0–686/10-s epoch); LPA (VM = 687–1,298/10-s epoch); and MVPA (VM ≥ 1,299/10-s epoch). Sixty minutes of consecutive zeros were considered non-wear time (47). To avoid selection bias, EY children were included irrespective of their attendance patterns (e.g., 15, 30 h, or full time), and attendance sessions typically lasted between 3 and 6 h/day. A minimum of 2 days of monitoring is recommended as necessary to achieve acceptable reliability for in-setting behaviour measurements in preschoolers (48). A 5-day data collection period was used to maximise reliability and account for variability in attendance, consistent with other early education PA research (49, 50).

Educators were asked to continue their normal practice, and a superhero analogy (that did not include mentioning PA) was introduced to assist with compliance. Superhero certificates were used as a motivational tool, provided to each participating child and setting at the end of the data collection period. On average, the participants’ (*n* = 201) total wear time was 1,419 min (SD = 529.15), equating to 23.6 h across the 5-day monitoring period per participant. PA data for two participants were excluded from PL and PA relationship analysis because of device battery failure and inadequate wear time. However, their PL data were used in the PL analysis. PA data were analysed using a custom MATLAB script. Total PA (TPA) was calculated as the combined accumulation of LPA and MVPA.

### PLEY Wheel tool

The PLEY Wheel is an assessment tool created to benchmark and score the skills, knowledge, and behaviours that a child is demonstrating across the affective, physical, cognitive, and social domains of PL. Twelve key components (Table 1) equally distributed across domains (three per domain) were selected through an iterative development process, informed by stakeholder top-ranked elements as identified as part of the England consensus statement consultation (51). Components were retained where they were considered to demonstrate contextual relevance and developmental appropriateness. Alternative structural configurations were considered, including a greater number of components per domain and unequal domain weighting. However, the 3-component-per-domain structure was selected to (1) ensure proportional representation, (2) support intuitive interpretation, and (3) limit time burden to complete. Indeed, shortened versions of the PL-C Quest (with 12-component items) have recently been developed and used with 8–12-year-olds (52) in response to the need for feasible and easy-to-administer assessments of PL. The PLEY Wheel tool components occupy equal segments of the wheel, with each scored using a five-point rating scale. A maximum total PL score of 60 (or 15 per domain) could be received, with the extent of shading on the PL Wheel indicating relative strengths or areas for development (Figure 1).

**Table 1:** The 12 components scored within the Physical Literacy Early Years (PLEY) wheel.

Domain	Component	Descriptor
Physical	1. Movement skills	The extent to which a child demonstrates movement skills involving different body parts across a variety of locomotor, stability, and object control activities
	2. Fitness	A child's ability to carry out daily movement and energetic games with vigour and alertness without undue fatigue, also considering overall physical health
	3. Competence	Ability to apply movement skills in various settings, contexts, and environments, as well as the ability to adapt during physical activities
Social	4. Relationships	The extent to which a child forms and maintains meaningful connections with others through physically active games, challenges, or tasks
	5. Social skills	Skills demonstrated during movement activities such as empathy and conflict resolution to verbal and non-verbal interactions
	6. Collaboration	Effective problem-solving during activity, contributing ideas or emotions and adapting to different contexts and audiences
Cognitive	7. Comprehension	The child's ability to grasp and comprehend simple movement terms and instructions, demonstrating proficiency in understanding and executing
	8. Understanding	The degree to which a child comprehends and interprets simple movement terms and concepts and can apply them in different contexts
	9. Knowledge	The extent to which a child demonstrates understanding of various activities, environments, or opportunities to be active
Affective	10. Confidence	To what extent the child seems confident in their abilities across a variety of different movement competencies and situations
	11. Motivation	How the child proactively takes part in energetic or active play, applying themselves to physical activity tasks with interest and enthusiasm
	12. Enjoyment	To what extent the child seems to enjoy experiences of participating in a variety of movement opportunities

Participating educators (*n* = 17) completed the tool by considering their familiarity, experience and observations of a child, and rating on the five-point scale where they consider the child for each of the 12 components. While educator-informed assessment introduces an element of subjectivity, this approach was intentionally adopted to prioritise ecological validity, feasibility, and application within real-world use. To mitigate bias, each component and rating level was supported by descriptors and real-world examples. A handout and 2-min instructional video were also provided, demonstrating how to complete the PLEY Wheel using an online platform developed for this study.

### Statistical analyses

Statistical tests were carried out using SPSS v29 (descriptive statistics, correlations and differences, and test–retest reliability), SPSS AMOS v29 (structural and construct validity) [IBM SPSS Statistics Inc., Chicago, IL, USA], and R v4.4.0 using the “psych” package (for internal consistency) (The R Foundation, Vienna, Austria). Statistical significance was set at *p* ≤ 0.05.

For study aim 1, a directed content analysis was used to evaluate face, content, and response processes. Confirmatory factor analysis (CFA) [a first-order CFA fitting measured variables to four hypothesised latent factors (PL domains) and a second-order CFA fitting latent variables to PL], composite reliability, convergent and discriminant validity statistics, intra-class correlations (ICC), and Polychoric Ordinal Alphas were used to establish evidence of structural and construct validity, test–retest reliability, and internal consistency, respectively. CFA fit indices indicating a good fit are given as follows: Comparative fit index (CFI), goodness-of-fit index (GFI), incremental fit index (IFI) > 0.90, adjusted goodness-of-fit index (AGFI) > 0.80, standardised root mean square residual (SRMR) < 0.08, and root mean square error of approximation (RMSEA) < 0.06 (53, 54). Factor loadings *λ* ≥ 0.4 were considered acceptable (55). ICCs were interpreted as poor (<0.50), moderate (0.50–0.75), good (0.75–0.90), and excellent (>0.90) (56). The factor loading for the first measured variable was fixed to 1, fixing the scale of the latent factor. Error terms from the measured variables were allowed to correlate if theoretically plausible. To determine whether the models differed based on sex and age, multi-group comparisons were made using configural and metric invariance tests.

For study aim 2, descriptive statistics were produced for demographic, socioeconomic/educational indicators, and PL and PA. Variables were tested for normality using the Kolmogorov–Smirnov and Shapiro–Wilk tests, and missing values were excluded from relevant analyses. Non-parametric analyses (Mann–Whitney *U*, Kruskal–Wallis, and Spearman's Rho correlations) were used to account for non-normally distributed variables and explore PL and PA differences by age, sex, IMD, BMI, and SEND. For non-parametric group comparisons, effect sizes were calculated using *r* (*z*/√*N*), while Spearman's rho (*ρ*) coefficients were used to quantify the strength and direction of associations. Correlation coefficients were interpreted as weak (0.10–0.29), moderate (0.30–0.49), or strong (≥0.50) (57). Bonferroni corrections were applied for multiple comparison. Linear mixed modelling was used to explore the PL–PA relationship, to account for setting-level effects. PA data were visualised using violin plots in Python 3 (Pandas, Seaborn, and Matplotlib).

For study aim 3, individual PL domain scores were converted into percentile ranks and categorised as low (<25th percentile), moderate (25th–75th percentile), or high (>75th percentile). A K-means cluster analysis was then conducted to identify natural groupings within the sample. A five-cluster solution was selected based on interpretability, convergence, and minimum distances between cluster centres. Quantitative indices (such as silhouette, elbow, or information criteria) are not generated when specifying a fixed number of clusters in K-means analysis, and therefore, a two-step cluster analysis was used to validate the optimal number of clusters by assessing conceptual distinctiveness and practical relevance. This included assessing each solution (3–6 clusters) manually using graphical outputs to evaluate conceptual distinctiveness, cluster quality, and practical relevance. Clusters were then labelled using a theory-informed approach aligned with multidimensional PL constructs. To support practical application, raw domain scores were mapped to low (3–6), moderate (7–11), or high (12–15) by considering mean, minimum, and maximum and where percentiles sit relative to raw scores. This created a simple profile assignment method. Strict, flexible, and total-score-based rules allocated all participants to one of the five PL profiles and enabled future profile assignment without rerunning percentile or cluster analyses. The conceptual strength and feasibility of this approach was evidenced by theoretical, empirical, and participant coverage statistics and mixed-methods content analysis.

## Results

Survey respondents (*n* = 90) were academic or research-based participants (*n* = 40), EY, PE, or sports educators (*n* = 38), strategic (managers, training, or policymakers, *n* = 27), and other (*n* = 4). A total of 61% of respondents were female (*n* = 55). Participants could select more than one professional role as applicable to them. A total of 72% (*n* = 65) of respondents selected England or the UK (including other home countries) as their country of work. A diverse worldwide geographical spread was represented in the remaining participants, which included Australia, Belgium, Canada, Finland, Germany, India, Indonesia, Latvia, Luxembourg, Poland, Portugal, Singapore, Sweden, Iran, Thailand, Turkey, and The Netherlands.

### PLEY Wheel face, content, and response process validity

Survey respondents (*n* = 90) rated the 12 PLEY Wheel items on a five-point scale (with five being “very important,” and one “not very important”). The mean rating scores ranged from 4.20 to 4.82, with enjoyment (*m* = 4.82, SD ± 0.38) and movement skills (*m* = 4.74, SD ± 0.44) the highest-rated items, and fitness the lowest (*m* = 4.20, SD ± 0.84). Further to this, 92% (*n* = 83) of survey respondents either strongly agreed or agreed that the 12 items included in the PLEY Wheel were relevant to the four PL domains. In relation to age, 85% (*n* = 77) strongly agreed or agreed that items were developmentally appropriate for 5-year-olds, 73% (*n* = 66) for 4-year-olds, and 64% (*n* = 58) for 3-year-olds. Qualitative feedback also emphasised this point. For example, survey respondent 40 (S40) commented, “I think all of the elements are appropriate once the child reaches the age of 5, however, some will be less relevant for 3-year-olds due to their overall development.” S7 questioned whether the verbal component may be harder for some younger children, and S84 added, “In my opinion, there is a significant difference between 3-year-olds and 5-year-olds across all measured aspects. The described characteristics align more closely with 5-year-olds and, to some extent, 4-year-olds, whereas 3-year-olds can still very much be like toddlers.” S13 added the reflection that “Perhaps for 3-year-olds there needs to be more nuanced, detailed indicators relevant to age and development as there may be more differences to capture at the younger age.” This was further reinforced by S27, who suggested that sequential and progressive descriptors for different ages might support this assessment.

Face-validity results show high user confidence and acceptance of the PLEY Wheel, with ratings across usability, clarity, language, measurement, and rating criteria summarised in Table 2. Overall, 97% of respondents (*n* = 87) were extremely or moderately confident that they understood how the PLEY Wheel is intended to be used, and 70% (*n* = 63) expressed that the tool accommodates individual developmental differences. Concerns were raised around inclusivity and contextualisation, particularly for neurodivergent or disabled children, and some questioned whether EY educators have the knowledge and understanding for judging some of the PLEY Wheel items.

**Table 2:** Mean ratings and agreement percentages for usability and face validity items.

Coverage type	Survey question	Mean (±SD)	% Agree/strongly agree (*n*)
Usability	The tool appears simple to use and not overly time-consuming	4.08 (± 0.93)	79 (*n* = 71)
Understanding	The PLEY wheel would be understood by EY practitioners	3.82 (± 0.89)	64 (*n* = 58)
Visual design	The visual design of the PLEY wheel effectively communicates its purpose	4.40 (± 0.73)	96 (*n* = 86)
Language	The language and terminology used in the PLEY wheel is appropriate	4.17 (± 0.77)	86 (*n* = 77)
Measuring physical literacy	The PLEY wheel appears to measure physical literacy in 3–5-year-olds	4.16 (± 0.76)	88 (*n* = 79)
Rating criteria	The rating criteria used in the PLEY wheel (rag rating 1–5) are appropriate	4.11 (± 0.80)	83 (*n* = 75)

The response processes of educators (*n* = 17, 88% female) were explored under the cognitive themes of “comprehension,” “retrieval,” “judgement,” and “response” to align with a commonly used four-stage cognitive model of assessment (58). The educators were nursery (65%, *n* = 11) or reception class (35%, *n* = 6) teachers. Summaries and illustrative educator quotes by theme are presented in Table 3.

**Table 3:** Summary of response process evidence.

Cognitive stage & summary	Illustrative participant quotes^a^
Comprehension Practitioners consistently demonstrated a clear understanding of each item's intent and the rating process, supported by video and descriptor guidance. Introductory training could further support first-time users	“The description of each criteria/question was detailed” (E1) “It was user-friendly, and video was helpful” (E5) “Before starting, a short training on what is expected as a teacher might have been useful” (E9) “Support from the researcher helped to ensure that I was able to use this easily” (E11) “Once I had completed the wheel for one child, I felt confident with what I should be doing” (E14) “Completing the wheel with the descriptors at hand was helpful to mark each child confidently” (E15)
Retrieval Responses indicated that practitioners drew primarily on direct observation and personal knowledge of the child, integrating multiple contexts (structured sessions and free play)	"It was interesting watching the interactions between children and how it changed and the dynamics, which also then impacted what was being seen” (E13) “I based my scoring on my own personal observations of the child in provision. (I work in the nursery for 2 out of 5 days, so I know the children well)” (E14) E15 mentioned that the process was “reflecting and watching their actions during a sports session and playing in garden and also discussing with other key persons in nursery” (E15) “I reflected on how the children participated in my PE lessons across a structured and free-play session so I could observe their responses to my instructions, how they embraced tasks and how they played with each other” (E16) To score the child I just thought about that child as a whole and how they are in sessions. If there was something I was unclear on I would go and observe the child and ask them to complete an activity or task with me” (E17)
Judgement Educators reported that the judgement process drew on both professional knowledge and contextual benchmarks, using the tool's descriptors, peer comparisons, comparative reasoning, and developmental expectations. All users felt they had sufficient familiarity to make informed judgments	“I thought about age related expectations (taken from birth to 5 and development matters) and how their individual skills matched the statements on the wheel. I thought about the children individually but then also in comparison with each other which enabled scaling them from 1-5” (E14) “5 would be a stand-out, something that you would immediately notice with the child or could never say they didn't do/ can't do, 4 would be areas where they are strong but have shown that the skill can wane sometimes. 3 would be 50/50, 2 would be show glimpses [of that skill] and 1 would never show it” (E15) “I used my judgement in reference to “sometimes, often, almost always, etc.” (E16)
Response The 1–5 scale was found to be workable. Completion of the wheel followed varying logical sequences (e.g., clockwise or handout descriptor order), and all confirmed the scale captured their intended judgements.	“I worked around the wheel clockwise, not sure why – it is just how my brain told me to do it, and then I checked the scores given for each area again before moving onto the next child” (E14) “The order was done on the order the supporting handout/descriptors were written (physical, social, cognitive, affective)” (E15). E16 noted the 1-5 options worked fine and that they “worked around the Wheel started at different points for different children” None were unsure about the rating “I was more unsure about making the observation correct. Having a proper understanding of each area more than the rating” (E15)

a Sample (*n* = 5), Educator participant coding = E13–E17.

### PLEY Wheel structural and construct validity

**Figure 2:**
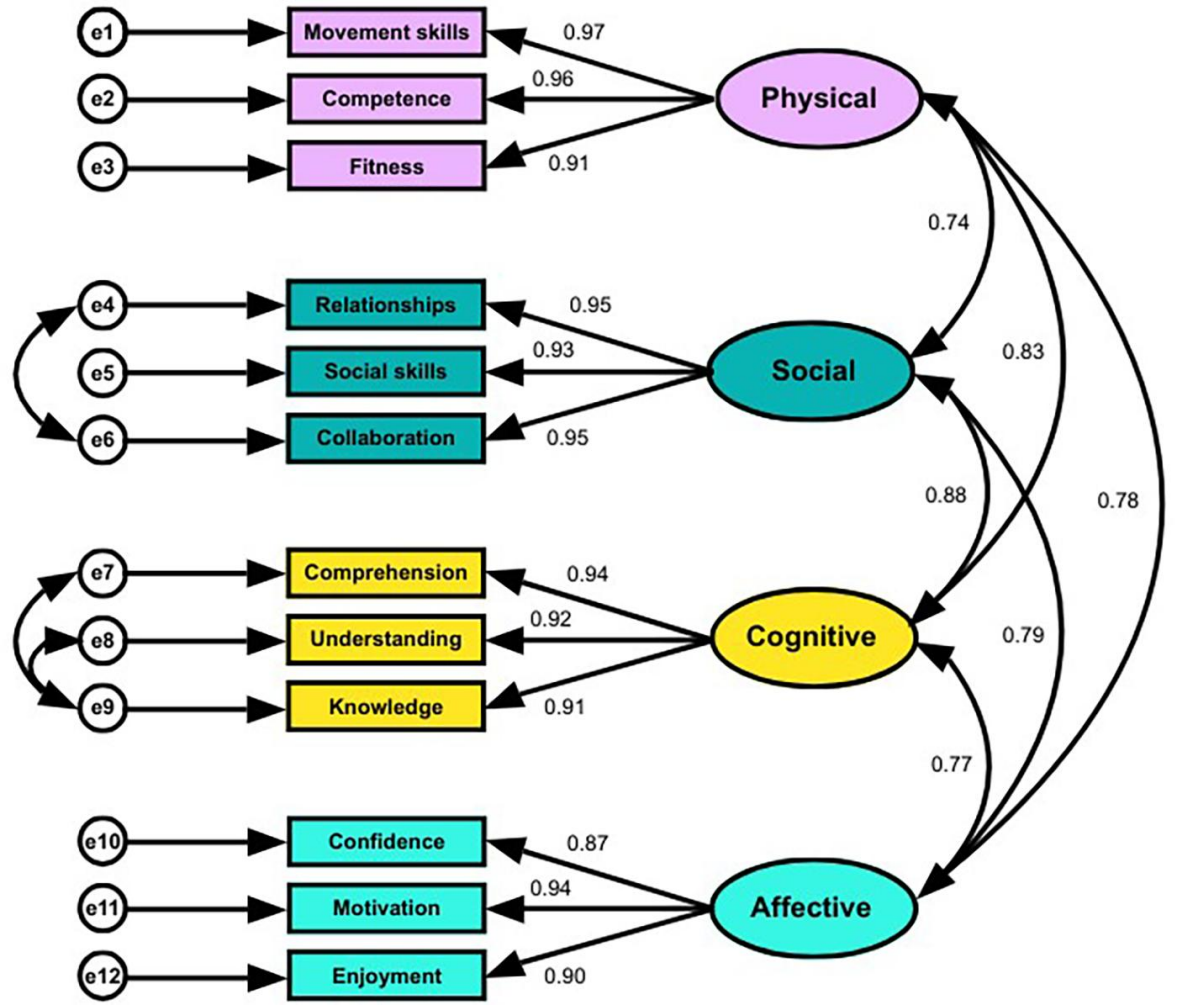
First-order confirmatory factor analysis (CFA) of the 12 measured items onto physical literacy domains.

Because of the nested nature of the data (practitioner level and child level), an ICC was conducted to see where the dependency lies. The overall single-measure ICC for PLEY Wheel scores (0.012) confirmed minimal practitioner-level variance and appropriateness for performing CFA at the child level to examine the structural validity of the PLEY Wheel. Goodness-of-model fit values for the first-order CFA (Figure 2) showed a good fit [CFI, 0.97; GFI, 0.91; AGFI, 0.85; RMSEA, 0.10; 90% CI 0.08–0.12; *χ*^2^(47) = 158.93, *p* < 0.001, *χ*^2^/df = 3.38], following additional correlations between error terms. Standardised loadings >0.7 indicate very strong item–factor relationships (51). All 12 items ranged between 0.87 and 0.97, evidencing strong loading onto the four hypothesised latent factors (PL domains). Each domain was positively and significantly correlated with the others (0.74–0.88), consistent with the conceptualisation of PL as an integrated construct with interrelated domains.

**Figure 3:**
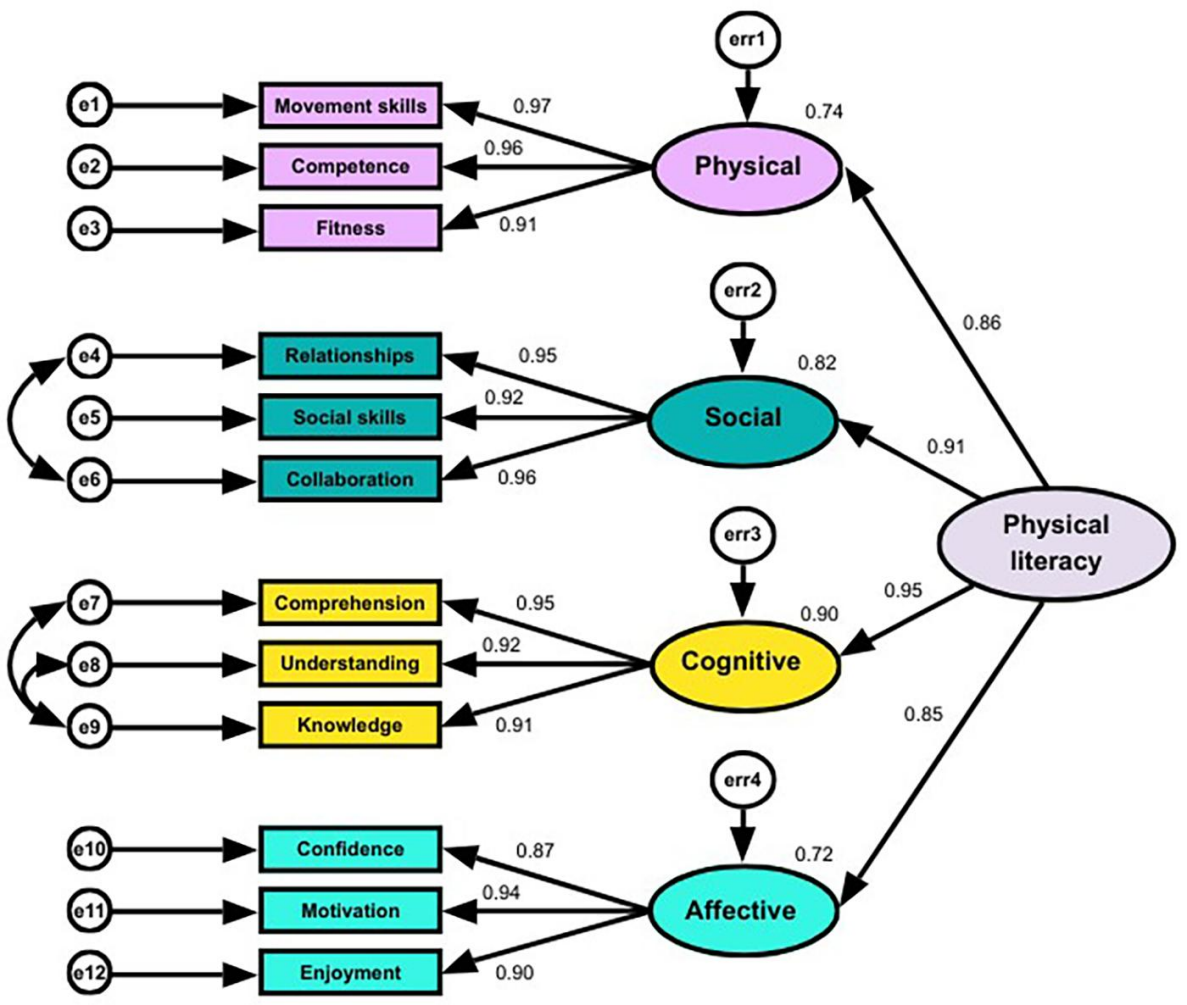
Second-order confirmatory factor analysis (CFA) of the four domains onto the construct of physical literacy.

The second-order model, with the four domains analysed as indicators of the overarching PL construct (see Figure 3), showed a good fit [CFI, 0.98; GFI, 0.92; AGFI, 0.86; RMSEA, 0.09; 90% CI 0.07–0.11; *χ*^2^(45) = 132.97, *p* < 0.001, *χ*^2^/df = 2.96]. Standardised loadings from the four domains ranged from 0.85 to 0.95, indicating that each domain contributed substantially to the overall PL construct. Additional multi-group CFA analyses and metric invariance testing showed no significant differences in terms of loading (all *p* > 0.05), chi-squared test, and model fit across sex and age (although the sample size was underpowered for age). In addition, all four domains demonstrated excellent compositive reliability (CR >0.7; CR = 0.93–0.96) and convergent validity (average variance extracted; AVE >0.5; AVE = 0.82–0.89). Discriminant validity was confirmed, with maximum shared variance (MSV) values lower than AVE and the square root of each construct's AVE exceeding inter-construct correlations, indicating that each PLEY Wheel domain captures a distinct but related aspect of PL.

### Test–retest reliability

For all participants (*n* = 31), the PLEY Wheel showed good to excellent test–retest reliability ICCs across total PL and the four domains, physical, social, cognitive and affective. The results from the stratified analysis revealed good reliability between sex and by age, and can be seen in Table 4. Only the affective domain among 3-year-olds (0.71, *p* = 0.002) showed moderate reliability (ICC between 0.5 and 0.75), with all others between 0.82 and 0.99. A follow-up paired-samples *t*-test for the moderate result revealed no significant difference between timepoints (*p* = 0.079), indicating that the moderate reliability likely reflects greater individual variability within this younger age group.

**Table 4:** Test–retest reliability results.

Scores	Group^a^	Test 1		Test 2		Test–retest		
		Mean	SD	Mean	SD	ICC	95% CI	*p*-Value
Physical literacy	All	35.58	13.16	35.52	15.06	0.94	0.88–0.97	<0.001
	Boys	36.25	13.92	34.06	17.02	0.93	0.80–0.97	<0.001
	Girls	34.87	12.74	37.07	13.06	0.96	0.82–0.99	<0.001
	Age 3	33.08	13.12	29.75	15.78	0.90	0.66–0.97	<0.001
	Age 4	37.85	13.95	40.00	14.91	0.97	0.85–0.99	<0.001
	Age 5	35.67	12.83	37.33	11.96	0.98	0.83–1	<0.001
Physical	All	8.90	3.35	8.87	3.99	0.89	0.79–0.95	<0.001
	Boys	9.31	3.44	8.44	4.59	0.90	0.70–0.96	<0.001
	Girls	8.47	3.31	9.33	3.33	0.90	0.64–0.97	<0.001
	Age 3	8.42	3.09	7.33	4.14	0.86	0.53–0.96	<0.001
	Age 4	9.54	3.36	10.15	3.65	0.90	0.71–0.97	<0.001
	Age 5	8.50	4.18	9.17	3.97	0.95	0.73–0.99	<0.001
Social	All	7.94	3.31	8.52	3.73	0.90	0.80–0.95	<0.001
	Boys	7.81	3.69	8.13	4.13	0.92	0.79–0.97	<0.001
	Girls	8.07	2.96	8.93	3.35	0.88	0.61–0.96	<0.001
	Age 3	7.33	3.68	7.33	3.96	0.90	0.69–0.97	<0.001
	Age 4	8.54	3.41	9.85	3.85	0.89	0.32–0.97	<0.001
	Age 5	7.83	2.48	8.00	2.28	0.99	0.92–1	<0.001
Cognitive	All	8.77	3.91	8.61	3.95	0.95	0.80–0.98	<0.001
	Boys	8.75	4.24	8.38	4.41	0.95	0.87–0.98	<0.001
	Girls	8.80	3.67	8.87	3.52	0.91	0.86–0.97	<0.001
	Age 3	7.67	4.03	6.83	3.90	0.90	0.68–0.97	<0.001
	Age 4	9.69	4.19	9.85	4.04	0.99	0.96–1	<0.001
	Age 5	9.00	2.97	9.50	2.88	0.95	0.72–0.99	<0.001
Affective	All	9.97	3.30	9.52	3.86	0.86	0.73–0.93	<0.001
	Boys	10.38	3.28	9.13	4.40	0.82	0.50–0.94	<0.001
	Girls	9.53	3.38	9.93	3.31	0.94	0.84–0.98	<0.001
	Age 3	9.67	3.03	8.25	4.07	0.71	0.26–0.90	0.002
	Age 4	10.08	3.50	10.15	3.63	0.95	0.86–0.99	<0.001
	Age 5	10.33	3.93	10.67	3.88	0.99	0.93–1	<0.001

a *n* = 31 (girls, *n* = 15; boys, *n* = 16; Age 3 years, *n* = 12; Age 4 years, *n* = 13; Age 5 years, *n* = 6).

### Internal consistency

Internal consistency for the full sample (*n* = 234) was examined through a polychoric correlation matrix in *R* (due to the ordinal nature of the data). From the matrix, a polychronic ordinal alpha (P*α*) was calculated for the overall data and for each of the four PL domains. Overall, P*α* = 0.98; physical (movement skills, fitness, and competence): P*α* = 0.98; social (relationships, social skills, and collaboration): P*α* = 0.97; cognitive (comprehension, understanding, and knowledge): P*α* = 0.97; affective (confidence, motivation, and enjoyment): P*α* = 0.96. When stratified by sex, the values for girls were as follows: overall, P*α* = 0.98; physical, P*α* = 0.99; social, P*α* = 0.97; cognitive, P*α* = 0.97; affective, P*α* = 0.95 and boys: overall, P*α* = 0.98; physical, P*α* = 0.98; social, P*α* = 0.98; cognitive, P*α* = 0.97; affective, P*α* = 0.96. Item-level correlations within domains were all strong (≥0.84), which supports that each item reliably measures its respective domain construct. Correlations between items from different domains were lower (0.63–0.79), reflecting the conceptual distinction between physical, social, cognitive, and affective domains.

### PLEY Wheel score characteristics and associations

Participant demographics and descriptive characteristics for phase 1 participants (*n* = 203) are presented in Table 5. A total of 76% of child participants were White British (*n* = 154), with 15 other ethnicity codes identified in the remaining 24% (*n* = 49). There were no significant differences in BMI; however, more girls (*n* = 18) classified as overweight or obese than boys (*n* = 10), and there was a higher proportion of boys with SEND and EAL.

**Table 5:** Participant demographics and descriptive characteristics.

Characteristic	All (*n* *=* 203) (mean ± SD)	Boys (*n* = 98) (mean ± SD)	Girls (*n* = 105) (mean ± SD)	Age 3^a^ (*n* *=* 71) (mean ± SD)	Age 4^a^ (*n* = 92) (mean ± SD)	Age 5^a^ (*n* = 40) (mean ± SD)
Age (years)	3.87 ± 0.72	3.87 ± 0.71	3.86 ± 0.73	–	–	–
Height (cm)	107.34 ± 6.54	108.19 ± 6.71	106.56 ± 6.32	101.97 ± 3.41	107.91 ± 5.26	114.79 ± 4.99
Mass (kg)	18.69 ± 3.40	18.92 ± 3.64	18.49 ± 3.17	16.72 ± 2.21	18.99 ± 3.23	21.28 ± 3.53
BMI (kg/m^2^)	16.14 ± 1.73	16.07 ± 1.75	16.20 ± 1.72	16.04 ± 1.44	16.24 ± 1.81	16.08 ± 1.99
BMI centile	57.45 ± 27.27	54.03 ± 28.42	60.54 ± 25.93	61.37 ± 24.82	55.88 ± 27.17	54.56 ± 31.12
Underweight BMI%	1.5	2.2	1.0	1.5	1.1	2.6
Healthy weight BMI%	80.3	87.0	81.4	86.2	83.3	82.1
Overweight BMI %	13.8	10.9	17.6	12.3	15.6	15.4
IMD decile	3.60 ± 2.92	3.46 ± 2.99	3.74 ± 2.99	2.85 ± 2.35	2.96 ± 2.02	6.28 ± 3.86
EAL %	13.4	15.3	11.5	19.7	12.1	5
SEND %	6.9	10.2	3.8	4.2	10.9	2.5
PPI %	10.4	7.9	12.9	16.2	9.5	2.5

a Age is measured in years. BMI, body mass index; IMD, index of multiple deprivation; EAL, English as an additional language; SEND, special educational needs or disabilities; PPI, pupil premium eligibility.

Table 6 presents the mean and standard deviation (mean ± SD) for total PL and its four subdomains (physical, social, cognitive, and affective) across the full sample (*n* = 234), stratified by sex and age.

**Table 6:** Physical Literacy Early Years (PLEY) wheel scores stratified by sex and age.

PL measure*	All (*n* = 234)(mean ± SD)	Boys (*n* = 114)(mean ± SD)	Girls (*n* = 120)(mean ± SD)	Age 3^a^ (*n* = 83)(mean ± SD)	Age 4^a^ (*n* = 105)(mean ± SD)	Age 5^a^ (*n* = 46)(mean ± SD)
Total PL	37.71 ± 11.01	36.59 ± 11.47	38.77 ± 10.49	36.51 ± 11.29	37.16 ± 10.84	41.11 ± 10.41
Physical	9.58 ± 2.98	9.34 ± 3.03	9.81 ± 2.92	9.17 ± 2.77	9.33 ± 2.83	10.89 ± 3.33
Social	8.96 ± 3.10	8.53 ± 3.27	9.37 ± 2.88	8.52 ± 3.21	9.16 ± 3.03	9.28 ± 3.01
Cognitive	9.17 ± 3.19	8.73 ± 3.28	9.59 ± 3.06	8.98 ± 3.32	8.84 ± 3.03	10.28 ± 3.12
Affective	10.00 ± 2.86	9.99 ± 3.02	10.00 ± 2.70	9.84 ± 2.90	9.83 ± 2.85	10.65 ± 2.76

* Total PL represents the sum of the four domain scores.

a Age is measured in years.

Total PL and domain scores aligned with expected developmental and demographic patterns. Girls scored higher than boys in both the social and the cognitive domains, with the largest differences observed in collaboration (*p* = 0.014, *r* = 0.173) and social skills (*p* = 0.034, *r* = 0.149). In the cognitive domain, girls scored significantly higher across all components: comprehension (*p* = 0.042, *r* = 0.143), understanding (*p* = 0.027, *r* = 0.155), and knowledge (*p* = 0.041, *r* = 0.144). Age-related differences were statistically significant, *H*(2) = 8.39, *p* = 0.015, *η^2^* = 0.041, illustrating an increase in PL by age. *Post hoc* tests showed no difference between 3- and 4-year-olds (*p* = 0.980), but 5-year-olds scored significantly higher than both 3- (*p* = 0.009, *r* = 0.25) and 4-year-olds (*p* = 0.007, *r* = 0.23). These differences were driven by the physical domain [*H*(2) = 15.72, *p* < 0.001] and cognitive domain [*H*(2) = 11.006, *p* = 0.004], particularly movement components (all *p* < 0.001) and cognitive components (*p* = 0.002–0.013). Domain correlations were the strongest in 3- and 4-year-olds, particularly physical–cognitive and social–cognitive (*ρ* = 0.848, *p* < 0.001 and *ρ* = 0.850, *p* < 0.001, respectively). At 5 years of age, interrelations became more moderate, cognitive–affective (*ρ* = 0.320, *p* = 0.044), and physical–social (*ρ* = 0.404, *p* = 0.010), although the physical–cognitive relationship remained large (*ρ* = 0.567, *p* < 0.001). Children with SEND had significantly lower total PL (*p* = 0.021, *r* = 0.162), physical (*p* = 0.008, *r* = 0.187), and cognitive scores (*p* = 0.003, *r* = 0.210). BMI was not significantly associated with total PL [*ρ* = –0.027; *H*(1) = 1.471, *p* = 0.225], although children categorised as overweight or obese had lower physical scores compared with their healthy weight peers [*H*(1) = 5.842, *p* = 0.016, *η^2^* = 0.026]. No differences in total PL were found across IMD tertiles [*H*(2) = 1.88, *p* = 0.391], although social domain scores varied [*H*(2) = 6.369, *p* = 0.041, *η^2^* = 0.022] and children from low-IMD settings scored lower in the physical domain (*p* = 0.031).

Across time in the ECEC setting, the average percentage of time spent in SB across our sample (*n* = 201) was 49% (SD **±** 8.30), which is equivalent to accumulating 29.4 min of TPA per hour (min/h). LPA averaged 29% (SD **±** 3.95, 17.4 min/h) and MVPA 22% (SD ± 6.05, 13.2 min/h). A summary of PA statistics stratified by setting is provided in Table 7.

**Table 7:** Descriptive statistics for physical activity.

Setting^a^	Month/year	Wear time^b^	% TPA (mean ± SD)	% SB (mean ± SD)	% LPA (mean ± SD)	% MVPA (mean ± SD)
1 R (*n* = 22)	June 24	1,914.99	54.33 ± 7.40	45.63 ± 7.41	29.32 ± 1.97	25.00 ± 7.13
2 N (*n* = 19)	July 24	1,240.54	54.37 ± 7.68	45.59 ± 7.70	32.57 ± 4.42	21.79 ± 5.40
3 N (*n* = 22)	September 24	1,716.60	52.06 ± 7.68	47.91 ± 7.69	28.77 ± 3.32	23.29 ± 5.70
4 N (*n* = 27)	October 24	572.33	57.79 ± 11.02	42.19 ± 11.03	32.78 ± 5.22	25.00 ± 8.22
5 NR (*n* = 13)	November 24	1,267.33	52.12 ± 6.95	47.85 ± 6.96	29.16 ± 3.33	22.96 ± 5.07
6 N (*n* = 21)	November 24	974.75	52.38 ± 3.95	47.59 ± 3.95	29.49 ± 3.32	22.90 ± 3.76
7 R (*n* = 16)	November 24	1,816.66	45.19 ± 5.35	54.78 ± 5.35	26.69 ± 2.56	18.50 ± 3.14
8 R (*n* = 34)	November 24	1,731.22	46.98 ± 6.76	52.98 ± 6.77	27.64 ± 3.01	19.34 ± 4.78
9 N (*n* = 15)	December 24	1,651.39	47.31 ± 7.72	52.66 ± 7.73	28.51 ± 3.15	18.81 ± 5.16
10 N (*n* = 13)	December 24	1,410.44	50.11 ± 6.42	49.86 ± 6.42	29.24 ± 3.17	20.86 ± 5.44

TPA, total physical activity; SB, sedentary behaviour; LPA, light physical activity; MVPA, moderate-to-vigorous physical activity.

a R, reception class; N, nursery class.

b Wear time=mean minutes per collection period.

**Figure 4:**
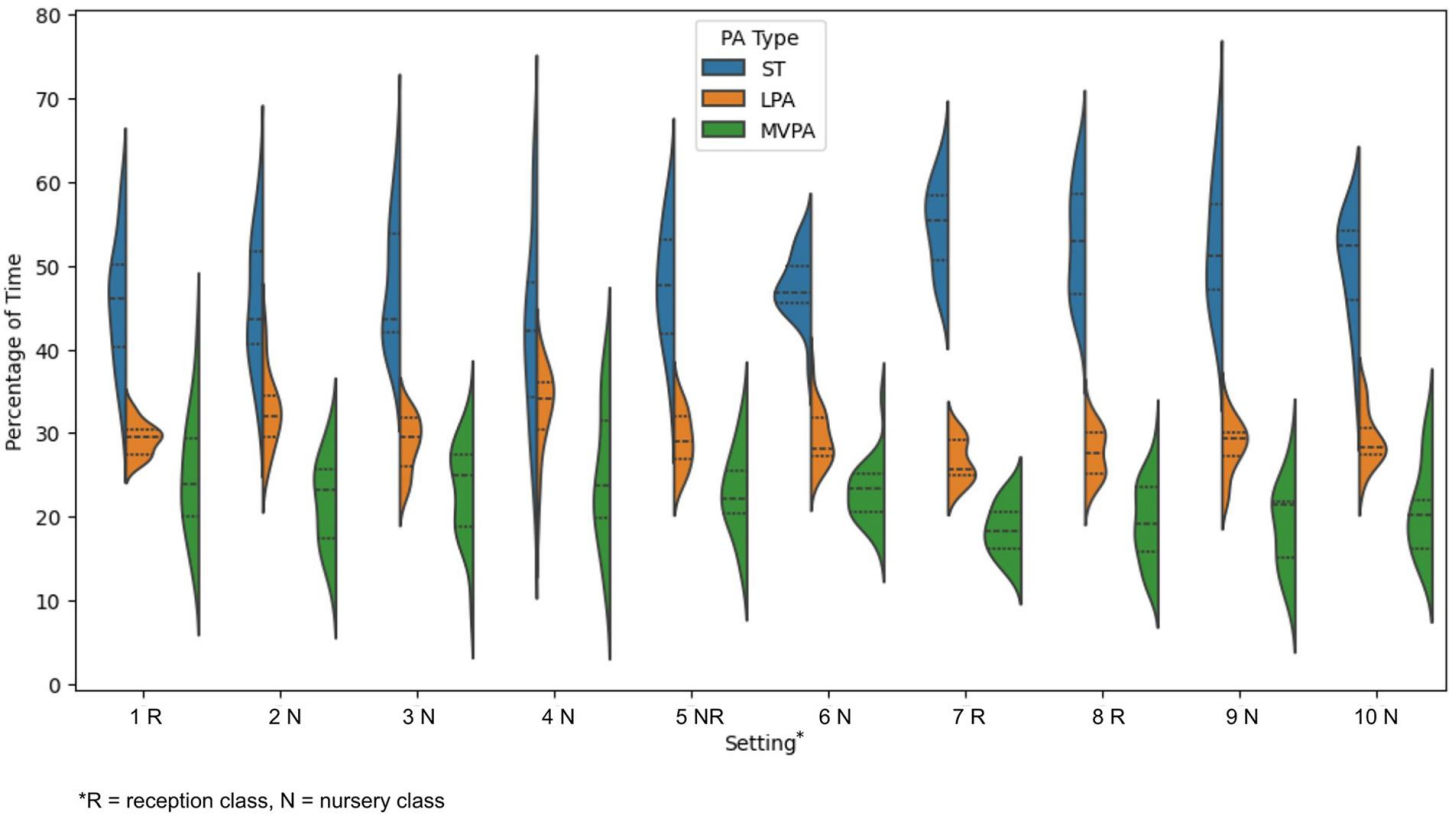
Setting level variation of sedentary (SB), light physical activity (LPA), and moderate-to-vigorous physical activity (MVPA).

There was significant PA variation between settings (all *p* < 0.001), and within a class, with effect sizes ranging from small to large (*η^2^* = 0.152–0.239). Overall, time spent per hour in SB ranged from 21.7% to 67.9% (13.0–40.7 min/h), LPA (18.2%–43.0%, 10.9–25.8 min/h), and MVPA (9.4%–41.5%, 5.6–24.9 min/h). Violin plots are presented in Figure 4 to illustrate the distribution and within-setting variability in time spent in SB, LPA, and MVPA.

Although non-significant (*U* = 4,622.0, *Z* = −1.02, *p* = 0.306, and *r* = −0.07), boys had higher MVPA (*M* = 22.8%, SD **±** 6.94, 13.7 min/h) than girls (*M* = 21.2%, SD **±** 5.01, 12.7 min/h), equivalent to one more minute of MVPA per hour. SB increased slightly and TPA decreased overall with age (age 3 years: *M* = 52.87%, SD **±** 8.38, 31.7 min/h; age 4 years: *M* = 50.87%, SD **±** 8.37, 30.5 min/h; age 5 years: *M* = 50.32%, SD **±** 7.81, 30.2 min/h). Children with SEND (*n* = 14) accumulated less TPA (47.68% vs. 51.73%) and engaged in lower MVPA (*M* = 19.67% vs. 22.14%), or 1.5 min/h less than children without SEND. TPA and MVPA across low (*n* = 153), medium (*n* = 28), and high (*n* = 22) IMD tertiles showed no significant differences between groups [*H*(2) = 4.72, *p* = 0.094], although a weak positive relationship was found, suggesting that children from higher IMD backgrounds engage in more MVPA, although this did not reach statistical significance (*ρ* = 0.05, *p* = 0.495). Similarly, there was a weak negative, but non-significant difference across BMI centile (*ρ* = −0.02, *p* = 0.815), and this difference was also not significant across weight groups, *H*(2) = 3.16, *p* = 0.206). To account for the clustering of children within the 10 settings, a linear mixed model was conducted to control for age and sex, with setting as a random effect. The results showed that PL (as scored by the PLEY Wheel) was significantly associated with TPA (*p* = 0.004). The model intercept of setting was significant (*p* < 0.001, *F* = 10,692.12), whereby settings accounted for a proportion of the variance in PA scores. Age (*p* = 0.074) and sex (*p* = 0.158) were non-significant as predictors.

Of particular note, the linear mixed model revealed certain points in the PL total score where predicted PA differed significantly. These occurred at PL = 37 (*p* = 0.010), PL = 46 (*p* = 0.017), PL = 52 (*p* = 0.013), and informed pragmatic total PL thresholds as follows: Very low <24 (14% *n* = 29), low 25–36 (31%, *n* = 62), moderate 37–44 (26%, *n* = 56), high (21%, *n* = 43), and very high (8%, *n* = 17). These thresholds were subsequently used within a rule-based approach to allocating children to physical literacy profiles. A negative association between TPA and the social domain (*ρ* = –0.140, *p* = 0.047) was found, driven by the social skills component (*ρ* = −0.157, *p* = 0.026).

### Novel profiling approach to PLEY Wheel scores

**Figure 5:**
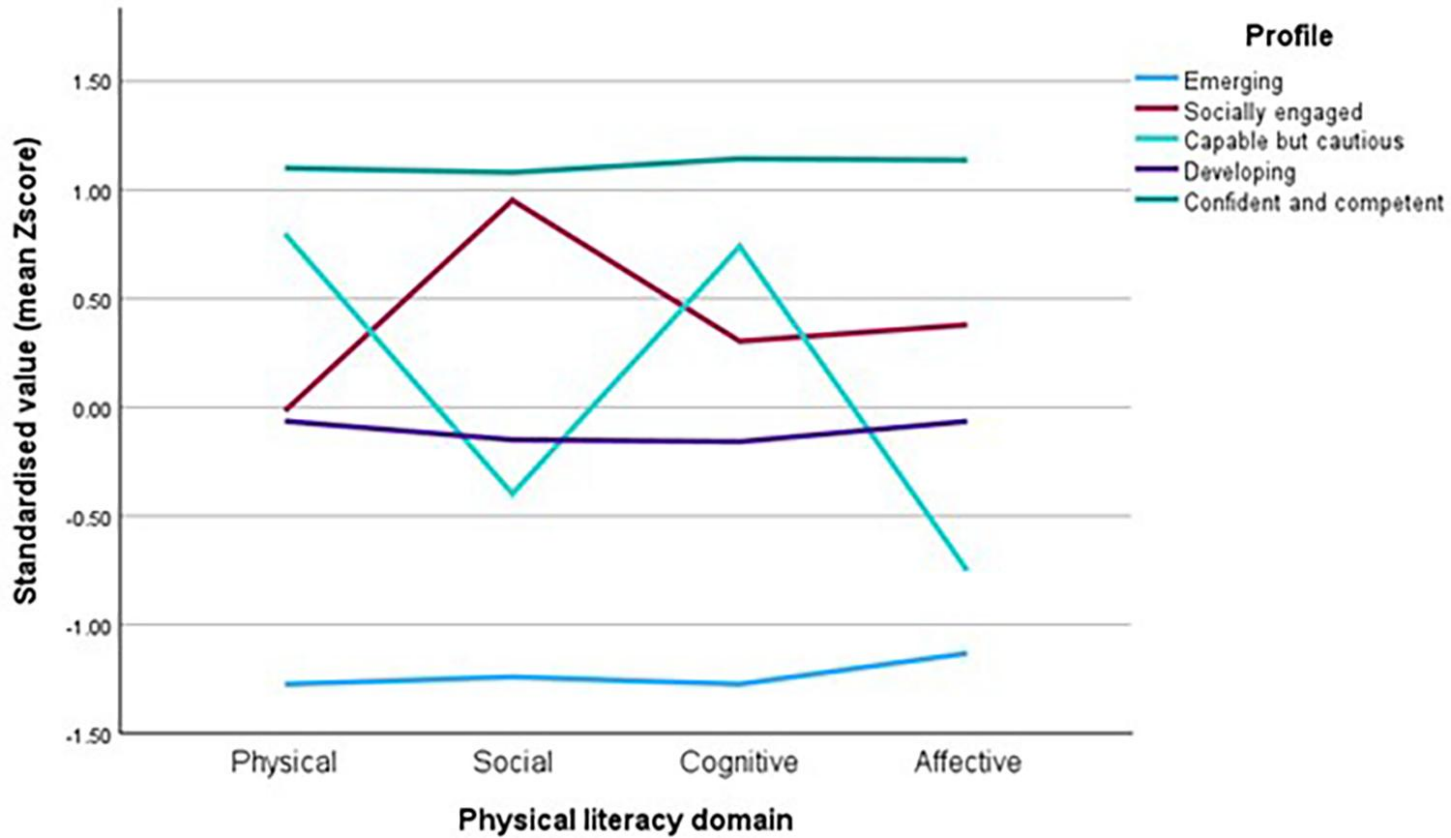
Standardised domain scores by profile.

PLEY Wheel total domain scores were converted to percentile ranks within the sample (*n* = 203) to establish children as low (<25th percentile), moderate (25th–75th), or high (>75th) and enable easily interpretable child-centred analyses of strengths and weaknesses across the four PL domains. Following a K-means cluster analysis, a five-cluster solution was selected, based on interpretability, convergence after four iterations, and minimum distances between cluster centres. The five profile groupings represent conceptually distinct configurations and were labelled as Emerging, Socially Engaged, Capable but Cautious, Developing, and Confident and Competent. Standardised domain scores illustrate these patterns across domains by profile (see Figure 5). Replicating the cluster analysis using raw scores confirmed the stability of the five-profile structure.

The score range for each domain was 3–15. Domain score distributions were sufficiently similar to apply common thresholds across domains (3–6 = low; 7–11 = moderate; 12–15 = high). Table 8 shows the five profiles mapped to raw scores. A rule-based approach was developed to allocate children to profiles, and a decision tree (see Supplementary Material 2) was created to assign children to profiles by answering a small set of guided questions, enabling practical use of the profiling approach without rerunning statistical analyses.

**Table 8:** Physical Literacy Early Years (PLEY) wheel profiling system.

Profile name	Physical^a^	Social^a^	Cognitive^a^	Affective^a^	Description
1. Emerging	3–6	3–6	3–6	3–6	Beginning to develop across all domains
2. Socially engaged	7–11	12–15	7–11	7–11	Thrives in social play, building confidence and competence
3. Capable but cautious	12–15	7–11	12–15	3–6	Physically able but less confident in engaging
4. Developing	7–11	7–11	7–11	7–11	Developing evenly across domains with room to grow
5. Confident and competent	12–15	12–15	12–15	12–15	Demonstrates strength across all aspects of PL development

a Domain score range = 3–15, bronze = low (score of 3–6), silver = moderate (score of 7–11), and gold = high (score of 12–15).

Of the 81 possible combinations of low, moderate, and high domain scores (based on three levels and four domains), the five-profile framework accounts for 67 combinations (82.7%). It also covers 32 out of 39 combinations present in the dataset (also 82.1%), reflecting strong conceptual design. Using the rule-based system, 87% of children were assigned to a profile using domain patterns alone; the remaining 13% were allocated using total PL score thresholds derived previously. The most common profile was “developing,” representing 40% (*n* = 81) of the sample, and the “capable but cautious” profile represented the least (5%, *n* = 10). Distribution by sex and age is given in Table 9.

**Table 9:** Physical literacy profile distribution summaries.

Profile	Total, *n* (%)	Girls, *n* (%)	Boys, *n* (%)	Age 3, *n* (%)	Age 4, *n* (%)	Age 5, *n* (%)
Emerging	44 (21.7)	17 (16.2)	27 (27.6)	14 (19.7)	25 (27.2)	5 (12.5)
Socially engaged	19 (9.4)	10 (9.5)	9 (9.2)	4 (5.6)	11 (12)	4 (10)
Capable but cautious	10 (4.9)	8 (7.6)	2 (2)	2 (2.8)	0 (0)	8 (20)
Developing	81 (39.9)	42 (40)	39 (39.8)	35 (49.3)	35 (38)	11 (27.5)
Confident and competent	49 (24.1)	28 (26.7)	21 (21.4)	16 (22.5)	21 (22.8)	12 (30)

No significant association was found between PL profile assignment and sex [*χ^2^* (4) = 6.80, *p* = 0.147]. Age was strongly associated with profile [*χ^2^ (*8) = 32.22, *p* < 0.001], with children aged 3- and 4 years more likely to be in the emerging and developing profiles (69%, *n* = 49 and 65.2%, *n* = 60, respectively) compared with 40% (*n* = 16) of 5-year-olds. Among 5-year-olds, a greater proportion were classified as confident and competent (30%, *n* = 12) and disproportionately represented the capable but cautious profile. Although the overall PL scores did not differ significantly by area-level deprivation, PL profile distribution varied significantly across IMD tertiles [*χ^2^* (8) = 45.07, *p* < 0.001]. Children from the most deprived group (low IMD tertile) were heavily concentrated in the emerging and developing profile (64.7%, *n* = 99). Linear mixed-effects modelling was used to examine whether profile-based representations of PL differentiated children's engagement in MVPA, accounting for clustering by setting and adjusting for age, sex, and IMD tertile. The MVPA model demonstrated an acceptable fit (AIC = 1,250.41) and was retained as the primary, policy-relevant outcome. Approximately 13% of the variance in MVPA was attributable to between-setting differences (ICC = 0.13), and the full model explained 19% of the variance in MVPA (conditional *R*^2^ = 0.19), indicating a modest explanatory power when both fixed and random effects were considered. Within this model, sex was the only significant predictor of MVPA, with girls accumulating significantly less MVPA than boys (*b* = −1.81, *p* = 0.031). In contrast, PL profile, age, and IMD tertile were not significantly associated with MVPA (all *p* > 0.25). Estimated marginal means for MVPA across the five PL profiles were similar, ranging from 20.9% to 23.7%, with overlapping 95% confidence intervals and no significant pairwise differences. These results suggest that, within this study sample, the PL profile alone does not strongly differentiate children's PA levels.

### Overall acceptability

Overall, the PLEY Wheel tool was considered very user-friendly or user-friendly by 90% of participating educators. In addition, 88% (*n* = 79) of survey respondents rated the PLEY Wheel tool as “excellent,” “very good,” or “good.” A total of 82% (*n* = 74) indicated that the tool could be applied beyond 5 years of age with (*n* = 60) or without adaptations (*n* = 14), suggesting perceived developmental flexibility. Qualitative responses further reinforced the value of the profiling approach, with respondents describing it as innovative, accessible, and supportive of PL development. For example, S43 described it as “an innovative step in the right direction for supporting and facilitating a positive PL journey at an impressionable age,” whilst another noted that “it has amazing potential” for use in practice (S35). Aligning with the England consensus statement definition, providing a shared language for discussing PL development, supporting communication with families, and steering intervention priorities were all factors that were valued. A minority of respondents expressed reservations about the use of fixed profiles, cautioning that categorisation may risk oversimplifying children's highly individual and PL journeys. Practicality and real-world constraints were also highlighted, such as time burden for whole-class completion. Whilst 10% (*n* = 9) believed that it would be “challenging” or “somewhat challenging” to implement the PLEY Wheel in typical ECEC settings, a strong evidence of demand, adaptation, and integration was also present. For example, respondents considered its use and age- and context-specific adaptation, also emphasising the importance of the tool fitting alongside, rather than replacing, existing assessment frameworks. To aid interpretation, feasibility responses were mapped onto established themes such as “acceptability,” “demand,” “implementation,” “practicality,” “adaptation,” and “integration” (39). Summaries and illustrative quotes by theme are presented in Table 10.

**Table 10:** Summary of feasibility evidence.

Feasibility measure & summary	Illustrative participant quotes^a^
Acceptability How practitioners react to the tool (e.g., clarity, perceived value). Responses highlighted the perceived value of the profiling approach in translating multidimensional PL data into accessible and actionable outputs. Respondents emphasised the clarity of the profiles and their utility for informing practice	“Physical literacy needs to be able to be understood and assessed by early years practitioners so any tool that supports this is a strength” (S4) It neatly distils a lot of numerical information into understandable categories, which can be used to drive tailored interventions” (S5) “Components that are often overlooked in training are measured” (S67)
Demand Anticipated or intended use and perceived need. Profiles were described as useful for supporting communication with families, providing a shared language to discuss PL development and anticipated routine use of the profiling outputs for planning, monitoring progress, and showing progress	“It would allow early years providers to share information with families as areas for development that can support families for ideas at home” (S88) “Enables settings to show progress. Individual work can be done with children and areas that need targeted work can be flagged and children may be grouped to work on specific areas” (S20) Clear guidance on developing independent learning plans that allow for progression on an individual basis (S7)
Implementation The extent, likelihood, and manner of delivery; automation vs manual use. There was recognition that this approach valued children's strengths, rather than focusing solely on deficits. There was strong support for a combination of automated profiling with practitioner oversight	“Automated will save loads of time… if you want practitioners to use this it cannot take too long” (S68) “A hybrid approach may be most effective where the tool offers automated suggestions based on observations entered, but the final decision remains with the educator” (S83) “Both manual and automated profiling have value. A hybrid approach—automated suggestions with practitioner review—offers the most reliable and context-sensitive solution” (S32) “Automated would save time but if practitioners are proficient with their PL and Early Years Foundation Stage (EYFS) knowledge, it would be good if they could add their own parameters and intervention” (S30)
Practicality Utilisation in practice; time, staffing, and workload constraints. The approach was generally viewed as straightforward, but the responses reflected variability in perceived added value, with some questioning duplication with existing assessment processes	"I found it a straight-forward process to complete” (E7) “A lengthy process for each child that didn't really give us any more information that we already knew from our internal assessments” (E17) “It is easy for staff to understand and gives a starting point on what might be areas of development or strengths” (S12) “Easy to use, quick and able to help the children who may need it as soon as possible” (S33) “I wonder how clear expectations will be of different ages. Having worked with lots of EY staff, sequential and progressive descriptors for different ages would enable consistency of assessment” (S28)
Adaptation Scope for modification across age, context, and inclusivity. The respondents highlighted the scope for adaptation across age groups, cultural contexts, and abilities. There was particular emphasis on the need for flexible descriptors and inclusive criteria to ensure relevance across diverse developmental and educational contexts	“The tool to me looks like it would fit for older children due to the terminology used. Or reduce the criteria to meet the needs of each age group and add the competency categories as they move through the age groups” (S75) “One way to improve intervention strategies is to include more culturally responsive and inclusive examples” (S83) “The absence of adaptive criteria or alternative demonstrations of competence can also leave educators guessing how to score children who communicate non-verbally, use mobility aids, or execute skills through modified techniques. Needs flexibly framed descriptors such as ‘selects an effective strategy suited to their body and environment”’ (S23) “The PLEY tool is measuring the children, how would a PLEY tool that measures the environment (programmes, pedagogical mindset/approach etc.) be designed” (S16)
Integration Fit with existing systems, assessments, and infrastructure. Viewed as feasible but conditional. The respondents emphasised that successful adoption would depend on clarity of purpose, time efficiency, training, and alignment with existing EYFS assessment structures, rather than positioning the tool as an additional burden	“This is a really easy to use guide that can sit alongside other assessment tools” (S82) “If they have the time to complete something like this, why they think they should complete it and how is it going to help improve their outcomes” (S53) “I think this would come down to the reality of how time consuming it is and the processes they have in place on translating the data into profiles and then assigning an intervention” (S74). “I think a ‘how to’ across a phased curriculum would be useful” (S62) “As with all educational tools. Ease of use is key…if teachers see it as an ‘add on’ they are less likely to use it” (S30) “Training would be required especially to map this to existing early learning assessment goals and the integration of this with other EY frameworks” (S6)

a Sample coding = survey respondent coding (S1–90), educator participant coding = E1–E17.

Implementation feasibility was further supported by quantitative responses for the PLEY profiling approach. A total of 87% (*n* = 78) “agreed” or “strongly agreed” that the profiles were easy to understand, meaningfully differentiated between children (76%, *n* = 68), supported interpretation of PL scores (71%, *n* = 64), and were named appropriately (69%, *n* = 62). In addition, the respondents provided feedback on suggested accompanying intervention strategies and identified areas for refinement, particularly in relation to inclusivity, activity examples, and implementation guidance.

## Discussion

In this study, content validity evidence demonstrates strong support for the relevance, importance, and clarity of the 12 items used within the PLEY Wheel, with high user confidence and positive feedback for the clear visual design and usable rating criteria. In particular, the CFA model evidenced strong item–factor relationships for all 12 component items to domains, with each domain positively and significantly correlated with each other. This is consistent with the conceptualisation of PL as an integrated construct with interrelated domains that are mutually reinforcing (59). The novel PLEY Wheel found significant age-related increases at 5 years, particularly in the physical and cognitive domains. This is in line with natural age-related growth (60) and motor development (61). Interestingly, inter-domain relationships were found to be the strongest at ages 3–4 and became more differentiated by age 5, particularly for the cognitive–affective and the physical–social domains. This indicates that as children mature, aspects of PL begin to differentiate rather than develop linearly. Given that PL development and practical application in early childhood remains underexplored (62), with emphasis on physical competence in assessment and intervention (63), there is a need for intervention content, goals, and outcomes to better cover all the core PL domains as children develop. In this study, class-related effects (e.g., that 4-year-olds could be in either nursery or reception) did not impact age-related increases in PL. Therefore, the differences likely reflect underlying age-related variation rather than independent class effects.

Specific PL domain scores generated by the PLEY Wheel showed sensitivity to age and sex, as well as variables such as SEND status, IMD tertile (deprivation), and weight classification. The finding that total PL was positively associated with the device-based measure of TPA, adds to the scant literature establishing the link between PL as a higher-order latent construct and PA (31). Empirical evidence of developmental differentiation between PL domains in early childhood is extremely limited; therefore, a novel aspect of this study was looking at domain-specific analysis and identifying data-led profile groups that enhance the understanding of how PL manifests in early childhood. Indeed, total PL metrics could mask important domain-level variations, as PL profiling analysis revealed socio-economic status as a significant influencer.

In addition, a negative association was observed between PA and the social domain of PL, driven by social skills. A negative association with MVPA when children are interacting has also been demonstrated (64). Given that girls scored higher in cognitive components as well as collaboration and social skills, and that sex was the only significant predictor of MVPA in profiling mixed model analysis (with girls accumulating significantly less MVPA than boys), differences in PL development and intervention approaches should be explored. However, overall, PL profiles were not significantly associated with MVPA, with the model intercept of setting accounting for a large proportion of the variance in PA. This aligns with the existing literature that environmental contexts (65), educator experience (66), and policy support (67) have a strong influence on PA behaviours in ECEC settings. The absence of strong profile-PA associations could be interpreted as evidence that PL and PA are related but distinct constructs. This is particularly relevant in relation to 3–5-year-olds in early childhood education, where the influence of environment context on behavioural outcomes such as PA is considerable (68). Indeed, in early childhood, PL may be more strongly related to how children engage, rather than how much they move.

Beyond psychometric performance, the PLEY Wheel demonstrates practical utility as a feasible, educator-facing assessment and reflection tool. Feasibility findings indicated strong support for its interpretability and visual design, supporting its application within early childhood education settings. Therefore, PLEY Wheel assessment and profiling are intended as flexible, child-centred, descriptive tools to support reflection and tailored intervention support based on needs; for example, confidence building, physical skill scaffolding, or collaborative tasks, rather than fixed classifications or predictors of future behaviour. Perceived developmental flexibility also highlights the potential for future extension and validation beyond EY contexts, including application within Key Stage 1 (5–7-year-olds). This would support a greater exploration of domain differentiation and enhance meaningful assessment and profile-based interpretation of PL.

### Strengths and limitations

This study is the first to provide validity and reliability evidence of a PL assessment tool that applies equal weighting across physical, social, cognitive, and affective domains for use in children aged 3–5 years of age. By integrating psychometric evaluation from a wide range of stakeholders, both nationally and internationally, the findings directly address the need for clearer, appropriate approaches to charting progress and assessment that aligns with the philosophical underpinnings of PL (69) and includes the social domain (70). The methodological variety and explicit and theory-informed examination of feasibility and interpretability alongside psychometric evaluation strengthen the study. In addition, the educator or class teacher has been deemed best placed to administer PL assessment in an educational context (37). Therefore, another important strength of this study is its multi-phased approach that includes educators (*n* = 17) who have used the PLEY Wheel, in addition to 42% of survey respondents being educators. A further strength is the use of device-measured PA, with accelerometers shown to be both valid and feasible for the assessment of free-living movement behaviour in this age group (71). Therefore, this study contributes to the picture of activity behaviours of young children, which is needed in ECEC settings (72).

Several limitations of this study should also be acknowledged. Perceived developmental appropriateness of the PLEY Wheel decreased with age (85%, 73%, and 64% for 5-, 4-, and 3-year-olds, respectively). Using a ≥70% agreement threshold, appropriateness for 3-year-olds did not meet this benchmark, with reflections that certain components may be less distinguishable at younger ages. Feedback identified whether age differentiation and language consistency could strengthen item definitions, alongside concerns related to educator knowledge, inclusive application, time constraints, and whether assessment could adequately capture the essence of PL. The debate on evaluating PL stems from the personalised, multidimensional nature of PL (73) and its extension beyond childhood (15). In addition, while settings varied in their characteristics, resources, and pedagogical approaches, it was beyond the scope of this study to map this detail to PL or PA. Similarly, this research did not extend into contexts outside of the ECEC setting, which could have strengthened interpretation of the PA and PL relationship.

Nonetheless, this study provides a robust empirical foundation for identifying, interpreting, and responding to domain-specific strengths and needs in early childhood. These findings highlight the importance of considering sex, developmental stage, and contextual factors when interpreting PL and designing interventions, as they influence both domain-specific strengths and engagement in PA. The nursery-to-reception transition presents a potentially important developmental window for targeted support, including developing teachers' skills to assess children's PL (28). Building on this work, future research should explore tailored intervention approaches aimed at all the core PL domains (74), with the PLEY Wheel offering potential applicability for assessing and supporting PL in children beyond 5 years of age. Finally, environmental, educator, policy, and further PL intervention impact research in this age group is recommended.

## Conclusion

The PLEY Wheel demonstrates strong evidence of structural and discriminant validity, good internal consistency and reliability, minimal rater effects, and alignment with theoretical expectations for PL. Acceptable feasibility and interpretability of the PLEY Wheel tool is also evidenced, confirming the PLEY Wheel as a reliable, theoretically grounded, and practically applicable tool for assessing PL in 3–5-year-old children in England. In addition, there is potential applicability beyond the current sample, supporting the future use of the PLEY Wheel with children over 5 years of age and in international contexts (subject to appropriate cultural adaptation and context-specific validation). While higher PL is positively associated with TPA, a deficit-based or “low”–“high” interpretation of PL, common in assessment, may mask important domain-level variations. Nonetheless, profile-based differences alone are insufficient to offset the dominant influence of setting, particularly in relation to MVPA. Even when boys and girls have similar PL profiles, they do not accrue MVPA equally. Therefore, the relationship between PL and PA may operate differently across domains and subgroups. For 3–4-year-olds, PL appears to develop as an integrated construct, before progressively differentiating across domains with age, highlighting a potential developmental window around 4–5 years of age. During this period, children's PL may plateau or develop unevenly across domains depending on opportunities, sex, socio-economic status, and educational need. Collectively, these findings suggest the nursery-to-reception transition as a pivotal point for targeted PL support. Finally, the PLEY Wheel and potential usefulness of the profiling approach present a promising direction for refinement, future resources, education, and bespoke intervention strategies aimed at more intentionally understanding and supporting PL development in early childhood.

## Data Availability

The raw data supporting the conclusions of this article will be made available by the authors, without undue reservation.

## Supplementary material

Supplementary Material 1 PLEY Wheel validation and feasibility survey.

Supplementary Material 2 Physical literacy early years (PLEY) Wheel decision tree.

## References

[1] Youth Sport Trust. Annual insight report. Available online at: https://www.youthsporttrust.org/researchlistings/research/pe-school-sport-the-annual-report-2023 (Accessed December 5, 2025).

[2] Department for Health and Social Care Physical Activity Guidelines. UK Chief Medical Officers’ Report. Available online at: https://www.gov.uk/government/publications/physical-activity-guidelines-uk-chief-medical-officers-report (Accessed 5, December 2025).

[3] Peers C Issartel J Behan S O’Connor N Belton S. Movement competence: association with physical self-efficacy and physical activity. Hum Mov Sci. (2020) 70:102582. 10.1016/j.humov.2020.102582 31957668

[4] Morales JS del Río EA Valenzuela PL Martínez-de-Quel Ó. Physical activity and cognitive performance in early childhood: a systematic review and meta-analysis of randomized controlled trials. Sport Med. (2024) 54:1835–50. 10.1007/s40279-024-02020-5 38598150

[5] Li D Wang D Zou J Li C Qian H Yan J Effect of physical activity interventions on children’s academic performance: a systematic review and meta-analysis’. Eur J Pediatr. (2023) 182:3587–601. 10.1007/s00431-023-05009-w 37227500

[6] Becker DR Nader PA. Run fast and sit still: connections among aerobic fitness, physical activity, and sedentary time with executive function during pre-kindergarten. Early Child Res Q. (2021) 57:1–11. 10.1016/j.ecresq.2021.04.007

[7] Department for Education. Giving every child the best start in life (2025). Available online at: https://www.gov.uk/government/publications/giving-every-child-the-best-start-in-life/giving-every-child-the-best-start-in-life (Accessed December 5, 2025).

[8] Durden-Myers EJ. Advancing physical literacy research in children. Children (Basel, Switzerland). (2024) 11(6):702. 10.3390/children11060702 38929281 PMC11202026

[9] International Physical Literacy Association. Definition of physical literacy. Available online at: https://www.physical-literacy.org.uk (Accessed December 5, 2025).

[10] Sport England. Physical literacy consensus statement for England. Available online at: https://www.sportengland.org/funds-and-campaigns/children-and-young-226 (Accessed December 5, 2025).

[11] Melby PS Nielsen G Brønd JC Tremblay MS Bentsen P Elsborg P. Associations between children’s physical literacy and well-being: is physical activity a mediator? BMC Public Health. (2022) 22(1):1267. 10.1186/s12889-022-13517-x 35768864 PMC9244357

[12] Ying Y Jiabin L Shanshan Z Kaixin Y Xiaomei L Shengyu W The relationship between physical literacy and physical activity: a systematic review and meta-analysis. Appl Psychol Health Well-Being. (2025) 17(4):e70062. 10.1111/aphw.70062 40760865

[13] Robinson LE Stodden DF Barnett LM Lopes VP Logan SW Rodrigues LP Motor competence and its effect on positive developmental trajectories of health. Sports Med. (2015) 45(9):1273–84. 10.1007/s40279-015-0351-6 26201678

[14] Hulteen RM Morgan PJ Barnett LM Stodden DF Lubans DR. Development of foundational movement skills: a conceptual model for physical activity across the lifespan. Sports Med. (2018) 48:1533–40. 10.1007/s40279-018-0892-6 29524160

[15] Whitehead M. Physical Literacy: Throughout the Lifecourse. London, UK: Routledge (2010).

[16] Parmar P Bika SL Pal S Halder S. Physical literacy as a catalyst for inclusive education in foundational stage aligns with NEP 2020. ShodhKosh J Visual Perform Arts. (2024) 5(1):175–86. 10.29121/shodhkosh.v5.i1.2024.3184

[17] Jurbala P. What is physical literacy, really? Quest. (2015) 67(4):367–83. 10.1080/00336297.2015.1084341

[18] Grauduszus M Wessely S Klaudius M Joisten C. Definitions and assessments of physical literacy among children and youth: a scoping review. BMC Public Health. (2023) 23:16680. 10.1186/s12889-023-16680-x PMC10486121 37679785

[19] Lafave LMZ Van Wyk N. Physical literacy from the start! The need for formal physical literacy education for early childhood educators. J Phys Educ Recreat Dance. (2024) 95(3):3–5. 10.1080/07303084.2024.2302755

[20] Weir N Pringle A Roscoe CMP. Physical literacy and physical activity in early years education: what’s known, what’s done, and what’s needed? Children. (2024) 11(11):1355. 10.3390/children11111355 39594930 PMC11593175

[21] Hurter L Essiet I Duncan M Roberts W Lewis K Goss H Physical Literacy Consensus for England: Evidence Review. London: Sport England (2022).

[22] Edwards LC Bryant AS Keegan RJ Morgan K Jones AM. Definitions, foundations and associations of physical literacy: a systematic review. Sports Med. (2017) 47(1):113–26. 10.1007/s40279-016-0560-7 27365029 PMC5215133

[23] Shearer C Goss H Edwards L Keegan R Knowles Z Boddy L How is physical literacy defined? A contemporary update. J Teach Phys Educ. (2018) 37:1–9. 10.1123/jtpe.2018-0136

[24] Lafave LMZ Van Wyk N Webster AD Hayek J Lafave MR. Validity and reliability of a physical literacy knowledge, attitudes, self-efficacy and behaviors questionnaire for early childhood educators (PLKASB-ECE): an exploratory factor analysis. PLoS One. (2024) 19(10):e0312736. 10.1371/journal.pone.0312736 39466825 PMC11516012

[25] Barratt J Dudley D Stylianou M Cairney J. A conceptual model of an effective early childhood physical literacy pedagogue. J Early Child Res. (2024) 22(3):381–94. 10.1177/1476718X231219580

[26] Liu Y Chen S. Physical literacy in children and adolescents: definitions, assessments, and interventions. Eur Phy Educ Rev. (2020) 27(1):96–112. 10.1177/1356336X20925502

[27] Diao Y Wang L Chen S Barnett LM Mazzoli E Essiet IA The validity of the Physical Literacy in Children Questionnaire in children aged 4 to 12. BMC Public Health. (2024) 24:869. 10.1186/s12889-024-18343-x 38515090 PMC10956319

[28] Barnett LM Jerebine A Keegan R Watson-Mackie K Arundell L Ridgers ND Validity, reliability, and feasibility of physical literacy assessments designed for school children: a systematic review. Sports Med. (2023) 53(10):1905–29. 10.1007/s40279-023-01867-4 37341907 PMC10504218

[29] Longmuir PE Boyer C Lloyd M Yang PH Boiarskaia E Tremblay MS. The Canadian Assessment of Physical Literacy: methods for children in grades 4 to 6 (8 to 12 years). BMC Public Health. (2018) 18(2):103. 10.1186/s12889-015-2106-6 26260572 PMC4532252

[30] Essiet IA Lander NJ Salmon J Duncan MJ Eyre EL Ma J A systematic review of tools designed for teacher proxy-report of children’s physical literacy or constituting elements. Int J Behav Nutr Phys Act. (2021) 18(1):131. 10.1186/s12966-021-01162-3 34620185 PMC8499583

[31] Cairney J Clark HJ James ME Mitchell D Dudley DA Kriellaars D. The preschool physical literacy assessment tool: testing a new physical literacy tool for the early years. Front Pediatr. (2018) 6:138. 10.3389/fped.2018.00138 29930933 PMC6001157

[32] Barnett LM Mazzoli E Hawkins M Lander N Lubans DR Caldwell S Development of a self-report scale to assess children’s perceived physical literacy. Phys Educ Sport Pedagogy. (2022) 27(1):91–116. 10.1080/17408989.2020.1849596

[33] Coombes L Bristowe K Ellis-Smith C Aworinde J Fraser LK Downing J Enhancing validity, reliability and participation in self-reported health outcome measurement for children and young people: a systematic review of recall period, response scale format, and administration modality. Qual Life Res. (2021) 30(7):1803–32. 10.1007/s11136-021-02814-4 33738710 PMC8233251

[34] Brown D Dudley DA Cairney J. Physical literacy profiles are associated with differences in children’s physical activity participation: a latent profile analysis approach. J Sci Med Sport. (2020) 23(11):1062–7. 10.1016/j.jsams.2020.05.007 32475780

[35] Cairney J Dudley D Kwan M Bulten R Kriellaars D. Physical literacy, physical activity and health: toward an evidence-informed conceptual model. Sports Med. (2019) 49(3):371–83. 10.1007/s40279-019-01063-3 30747375

[36] Department for Education. Childcare and early years survey of parents 2024. Available online at: https://explore-education-statistics.service.gov.uk/find-statistics/childcare-and-early-years-survey-of-parents./2024 (Accessed December 5, 2025).

[37] Goss HR Shearer C Knowles ZR Boddy LM Durden-Myers EJ Foweather L. Stakeholder perceptions of physical literacy assessment in primary school children. Phys Educ Sport Pedagogy. (2022) 27(5):515–30. 10.1080/17408989.2021.1911979

[38] Farooq MA Parkinson KN Adamson AJ Pearce MS Reilly JK Hughes AR Timing of the decline in physical activity in childhood and adolescence: Gateshead Millennium cohort study. Br J Sports Med. (2018) 52(15):1002–6. 10.1136/bjsports-2016-096933 28288966 PMC6204977

[39] Simpson A Stein M Rosenberg M Ward B Derbyshire A Thornton AL Early childhood educator outcomes from online professional development for physical literacy: a randomised controlled trial. Psychol Sport Exerc. (2023) 68:102464. 10.1016/j.psychsport.2023.102464 37665906

[40] Mokkink LB Terwee CB Patrick DL Alonso J Stratford PW Knol DL The COSMIN checklist for assessing the methodological quality of studies on measurement properties of health status measurement instruments: an international Delphi study. Qual Life Res. (2010) 19(4):539–49. 10.1007/s11136-010-9606-8 20169472 PMC2852520

[41] NHS Digital. Calculate body mass index (BMI) for children and teenagers. Available online at: https://www.nhs.uk/health-assessment-tools/calculate-your-body-mass-index/calculate-bmi-for-children-teenagers (Accessed December 5, 2025).

[42] Tyler R Miller C Barnett LM Fairclough SJ MacDonald MJ. Validity and reliability of the Physical Literacy in Children Questionnaire (PL-C Quest) for primary school children aged 8–11 years. J Sci Med Sport. (2025) 28(6):483–90. 10.1016/j.jsams.2025.03.010 40180850

[43] Migueles JH Cadenas-Sanchez C Ekelund U Delisle Nyström C Mora-Gonzalez J Löf M Accelerometer data collection and processing criteria to assess physical activity and other outcomes: a systematic review and practical considerations. Sports Med. (2017) 47(9):1821–45. 10.1007/s40279-017-0716-0 28303543 PMC6231536

[44] Pate RR Davis MG Robinson TN Stone EJ McKenzie TL Young JC. Promoting physical activity in children and youth: a leadership role for schools. Circulation. (2006) 114(11):1214–24. 10.1161/CIRCULATIONAHA.106.177052 16908770

[45] Fairclough SJ Noonan R Rowlands AV Van Hees V Knowles Z Boddy LM. Wear compliance and activity in children wearing wrist- and hip-mounted accelerometers. Med Sci Sports Exerc. (2016) 48(2):245–53. 10.1249/MSS.0000000000000771 26375253

[46] Dobell A Pringle A Faghy MA Roscoe CMP. Fundamental movement skills and accelerometer-measured physical activity levels during early childhood: a systematic review. Children. (2020) 7(11):224. 10.3390/children7110224 33187252 PMC7697076

[47] Byun W Beet MW Pate RR. Sedentary behavior in preschoolers: how many days of accelerometer monitoring is needed? Int J Environ Res Public Health. (2015) 12(10):13148–61. 10.3390/ijerph121013148 26492261 PMC4627022

[48] Troiano RP. Large-scale applications of accelerometers: new frontiers and new questions. Med Sci Sports Exerc. (2007) 39:1501. 10.1097/mss.0b013e318150d42e 17805080

[49] Pate RR O’Neill JR Byun W McIver KL Dowda M Brown WH. Physical activity in preschool children: comparison between Montessori and traditional preschools. J Sch Health. (2014) 84:716–21. 10.1111/josh.12207 25274171 PMC4185392

[50] Maishman R Dobell A Pallan M Blair PS White J Simpson SA Accelerometer-measured physical activity in UK early childhood education and care settings: a cross-sectional study. J Phys Act Health. (2025) 1(AOP):1–9. 10.1123/jpah.2025-0316 41285124

[51] Duncan MJ Essiet I Hurter L Roberts WM Lewis K Goss H Stakeholder perceptions of physical literacy: results from a national consultation in England. Front Sports Act Living. (2024) 6:1457845. 10.3389/fspor.2024.1457845 39483958 PMC11524871

[52] Carl J Mazzoli E De Silva C Herfet M Arundell L Sahlqvist S Deriving and validating a short version of the physical literacy in children questionnaire. J Sci Med Sport. (2025):S1440-2440(25)00528-6. 10.1016/j.jsams.2025.11.018 41421885

[53] Wang L Fan X Wilson VL. Effects of non-normal data on parameter estimates and fit indices for a model with latent and manifest variables: an empirical study. Struct Equ Modeling. (1996) 278(3):228–47. 10.1080/10705519609540042

[54] Hu L-T Bentler PM. Cutoff criteria for fit indexes in covariance structure analysis: 280 conventional criteria versus new alternatives. Struct Equ Modeling. (1999) 6(1):1–55. 10.1080/10705519909540118

[55] Hair JF Anderson RW Tatham RL Anderson RE Black WC. Multivariate Data Analysis. 5th Ed. London: Prentice-Hall (1998).

[56] Coo TK Li MY. A guideline of selecting and reporting intraclass correlation coefficients for reliability research. J Chiropr Med. (2016) 15(2):155–63. 10.1016/j.jcm.2016.02.012 27330520 PMC4913118

[57] Cohen J. Statistical Power Analysis for the Behavioral Sciences. 2nd ed. Hillsdale, NJ: Lawrence Erlbaum Associates, Publishers (1988).

[58] Tourangeau R. Cognitive science and survey methods: a cognitive perspective. In: Jabine T Straf M Tanur J Tourangeau R, editors. Cognitive Aspects of Survey Methodology: Building a Bridge Between Disciplines. Washington, DC: National Academy Press (1984). p. 73–100.

[59] Valle-Muñoz VM Mendoza-Muñoz M Villa-González E. Physical literacy as a pedagogical model in physical education. Children (Basel, Switzerland). (2025) 12(8):1008. 10.3390/children12081008 40868461 PMC12384994

[60] Roscoe CMP Taylor N Weir N Flynn RJ Pringle A. Impact and implementation of an early years fundamental motor skills intervention for children 4–5 years. Children (Basel). (2024) 11(4):416. 10.3390/children11040416 38671633 PMC11048878

[61] Barnett LM Lai SK Veldman SL Hardy LL Cliff DP Morgan PJ Correlates of gross motor competence in children and adolescents: a systematic review and meta-analysis. Sports Med. (2016) 46:1663–88. 10.1007/s40279-016-0495-z 26894274 PMC5055571

[62] Wilhite BC Chui K Sacheck JM Hatfield DP Morris M Ziembowicz M Getting an active start: assessing the impact of a physical literacy-based intervention on preschool-aged children’s fundamental movement skills, motor competency and behavioral self-regulation. Int J Environ Res Public Health. (2025) 22(12):1861. 10.3390/ijerph22121861 41464494 PMC12732799

[63] Carl J Barratt J Töpfer C Cairney J Pfeifer K. How are physical literacy interventions conceptualized?—a systematic review on intervention design and content. Psychol Sport Exerc. (2022) 58:102091. 10.1016/j.psychsport.2021.102091

[64] Woodfield L Tatton A Myers T Powell E. Predictors of children’s physical activity in the early years foundation stage. J Early Child Res. (2021) 20(2):199–213. 10.1177/1476718X211052797

[65] Vanderloo LM Tucker P Johnson AM Burke SM Irwin JD. Environmental influences on preschoolers’ physical activity levels in various early-learning facilities. Res Q Exerc Sport. (2015) 86(4):360–70. 10.1080/02701367.2015.1053105 26288191

[66] Mazzucca S Hales D Evenson KR Ammerman A Tate DF Berry DC Physical activity opportunities within the schedule of early care and education centres. J Phys Act Health. (2018) 15(2):73–81. 10.1123/jpah.2017-0071 28872405

[67] Lum M Grady A Falkiner M Jones J Finch M Green S Assessing the implementation of healthy eating and physical activity policies and practices in the family day care setting: a cross-sectional study. Health Promot J Austr. (2021) 32(S2):116–25. 10.1002/hpja.420 32945037

[68] Machado-Rodrigues AM Rodrigues D Gama A Nogueira H Silva MG Mascarenhas LP Objectively measured sedentary time and physical activity levels in a sample of pre-school children: amounts and obesity risk. Minerva Pediatrics. (2021) 77(5):425–30. 10.23736/S2724-5276.21.06584-8 34859649

[69] Green NR Roberts WM Sheehan D Keegan RJ. Charting physical literacy journeys within physical education settings. J Teach Phys Educ. (2018) 37:272–9. 10.1123/jtpe.2018-0129

[70] Edwards LC Bryant AS Keegan RJ Morgan K Cooper SM Jones AM. Measuring’ physical literacy and related constructs: a systematic review of empirical findings. Sports Med. (2018) 48:659–82. 10.1007/s40279-017-0817-9 29143266 PMC5808047

[71] Phillips SM Summerbell C Hobbs M Hesketh KR Saxena S Muir C A systematic review of the validity, reliability, and feasibility of measurement tools used to assess the physical activity and sedentary behaviour of pre-school aged children. Int J Behav Nutr Phys Act. (2021) 18(1):141. 9. 10.1186/s12966-021-01132-9 34732219 PMC8567581

[72] O’Brien KT Vanderloo LM Bruijns BA Truelove S Tucker P. Physical activity and sedentary time among preschoolers in centre-based childcare: a systematic review. Int J Behav Nutr Phys Act. (2018) 15:117. 10.1186/s12966-018-0745-6 30463585 PMC6249856

[73] Robinson DB Randall L. Marking physical literacy or missing the mark on physical literacy? A conceptual critique of Canada’s physical literacy assessment instruments. Meas Phys Educ Exerc Sci. (2017) 21(1):40–55. 10.1080/1091367X.2016.1249793

[74] Kwan MYW Graham JD Bedard C Bremer E Healey C Cairney J. Examining the effectiveness of a pilot physical literacy–based intervention targeting first-year university students: the PLUS program. SAGE Open. (2019) 9(2):2158244019850248. 10.1177/2158244019850248

