# Supplementary material for: Advancing physical literacy measurement in early childhood: psychometric properties of a novel assessment and profiling method and its relationship with physical activity
Source: Front Sports Act Living. 2026 Mar 19;8:1773645. doi: 10.3389/fspor.2026.1773645 (PMC13044117; doi:10.3389/fspor.2026.1773645)
Supplement: Supplementary Material 1 — PLEY Wheel validation and feasibility survey. [file Table1.docx]

**PLEY Wheel validation and feasibility survey**

Qu 1. Please read the research information letter and confirm you provide your consent to take part in this research

Yes * [required to continue]

No

Qu 2. Name

Qu 3. Email address

Qu 4. We will use a unique identification code you analyse and report your responses. Please provide the last two letters of your postcode and last two numbers of your telephone number below (e.g. NA03)

Qu 5. In which capacity are you currently working? (Select all that apply)

Academic/Researcher

Early Years Practitioner

PE or Sport Educator

Strategic (e.g. active partnership, local authority, training, policy)

Other

Qu 6. In which country do you currently work in?

Qu 7. How many years of experience do you have in the following areas?

[Can select <2 years, 2-5 years, 6-10 years and 10+ years]

Physical literacy

Physical activity

Early childhood physical development

Education

Qu 8. Considering your professional experience, on a scale of 1 to 5 (1 being minimal and 5 being extensive), how would you rate your familiarity and practical application of physical literacy [select from 1 – 5 stars]

Qu 9. Prior to completing this section, please watch this 2-minute video providing an overview and instructions on how to complete the PLEY wheel  <https://vimeo.com/981836474/7064ae16c9?share=copy>

[Image of the PLEY Wheel shown]

The tool asks educators to select scores on a 5 point scale across 12 components. This quantifies the skills, knowledge and behaviours that a child is currently demonstrating within the 4 domains of physical literacy: physical (how they move), social (how they connect), cognitive (how they think) and affective (how they feel). The maximum total score possible using this tool is 60, equally weighted (5 per question, 15 per domain). How confident are you that you understand how the PLEY wheel is intended to be used?

Extremely confident

Very confident

Moderately confident

Slightly confident

Not at all confident

Qu 10. Please rate the importance of each of the 12 components used on the PLEY Wheel

[Can select very important, important, moderately important, slightly important or not important]

Movement skills

Fitness

Competence

Relationships

Social skills

Collaboration

Knowledge

Understanding

Comprehension

Confidence

Motivation

Enjoyment

Qu 11. In your view, are the component definitions clearly defined and easy to understand?

Yes

No

Qu 12. If no, explain why not?

Qu 13. After seeing the PLEY Wheel, please rate your agreement with the following statements

[Can select Strongly agree, Agree, Neutral, Disagree, Strongly disagree]

The PLEY Wheel appears to measure physical literacy in 3-5-year-olds

The components included in the PLEY Wheel are relevant to the physical literacy domains

The language and terminology used in the PLEY Wheel is appropriate

The PLEY Wheel would be understood by early years practitioners

The visual design of the PLEY Wheel effectively communicates its purpose

The tool appears simple to use and not overly time consuming

Qu 14. Please rate your agreement with the following statements

[Can select Strongly agree, Agree, Neutral, Disagree, Strongly disagree]

The components measured in the PLEY Wheel are developmentally appropriate for all 3-year-olds

The components measured in the PLEY Wheel are developmentally appropriate for all 4-year-olds

The components measured in the PLEY Wheel are developmentally appropriate for all 5-year-olds

The PLEY Wheel adequately accommodates for individual differences in development

The rating criteria used in the PLEY Wheel (rag rating 1-5) is appropriate

Qu 15. If you disagreed with any of the statements above, please explain your reasoning or suggest how it could be improved

Qu 16. Feedback from educators who have used the PLEY Wheel in practice has indicated a generally positive experience, with 9 out of 10 rating it as user-friendly or very user-friendly with an estimated 5 mins per child time requirement for completion. In your opinion, how feasible would it be to implement the PLEY Wheel in typical early years settings?

Very feasible

Feasible with some support

Somewhat challenging

Challenging

Not feasible

Qu 17. What support would practitioners need to effectively use the PLEY Wheel? (Select all that apply) *

Training workshops

Video guides

Written examples

One-to-one support

Online support

Other

Qu 18. Overall, how would you rate the PLEY wheel as a tool for quantifying physical literacy in early years children?

Excellent

Very good

Good

Fair

Poor

Qu 19. In your opinion, could the PLEY Wheel be useful for assessing physical literacy in children older than 5 years?

Yes, with no changes

Yes, but with adaptations

No, not suitable for older children

Not sure

Qu 20. A profiling approach to physical literacy. Before completing this final section - please click and review this PL profiles document <https://tinyurl.com/yprndntc>

This proposes five different PL profiles in application to early years children (driven by real data created from over 200 sets of PLEY Wheel scores) and suggested intervention strategies for each profile group

After you have reviewed the PL profiles document, please rate your agreement with the following statements

[Can select Strongly agree, Agree, Neutral, Disagree, Strongly disagree]

These profiles will allow us to meaningfully differentiate between children with different PL levels

The profiles shown are easy to understand and interpret

The scoring criteria for assigning children to different profiles is appropriate

The profile names are appropriate

The targeted intervention approaches suggested are appropriate

Qu 21. What strengths do you see in this profiling approach?

Qu 22. How well do the five profile descriptions and characteristics match what you observe in early years children?

Extremely well

Very well

Moderately well

Slightly well

Not well at all

Qu 23. How helpful do you find the suggested intervention strategies for each profile?

Extremely helpful

Very helpful

Moderately helpful

Slightly helpful

Not helpful at all

Qu 24. Do you have suggestions for how the intervention strategies could be improved or expanded?

Qu 25. Would you prefer to assign children to profiles using:

A decision tree or visual guide

An automated scoring system (software)

A checklist-style rubric with scoring guide

I’m not sure

Qu 26. Finally, do you have any additional comments regarding whether practitioners should assign profiles manually, or be automated (e.g. within the PLEY Wheel online tool)?
