# Supplementary figures and images for: Advancing physical literacy measurement in early childhood: psychometric properties of a novel assessment and profiling method and its relationship with physical activity
Source: Front Sports Act Living. 2026 Mar 19;8:1773645. doi: 10.3389/fspor.2026.1773645 (PMC13044117; doi:10.3389/fspor.2026.1773645)

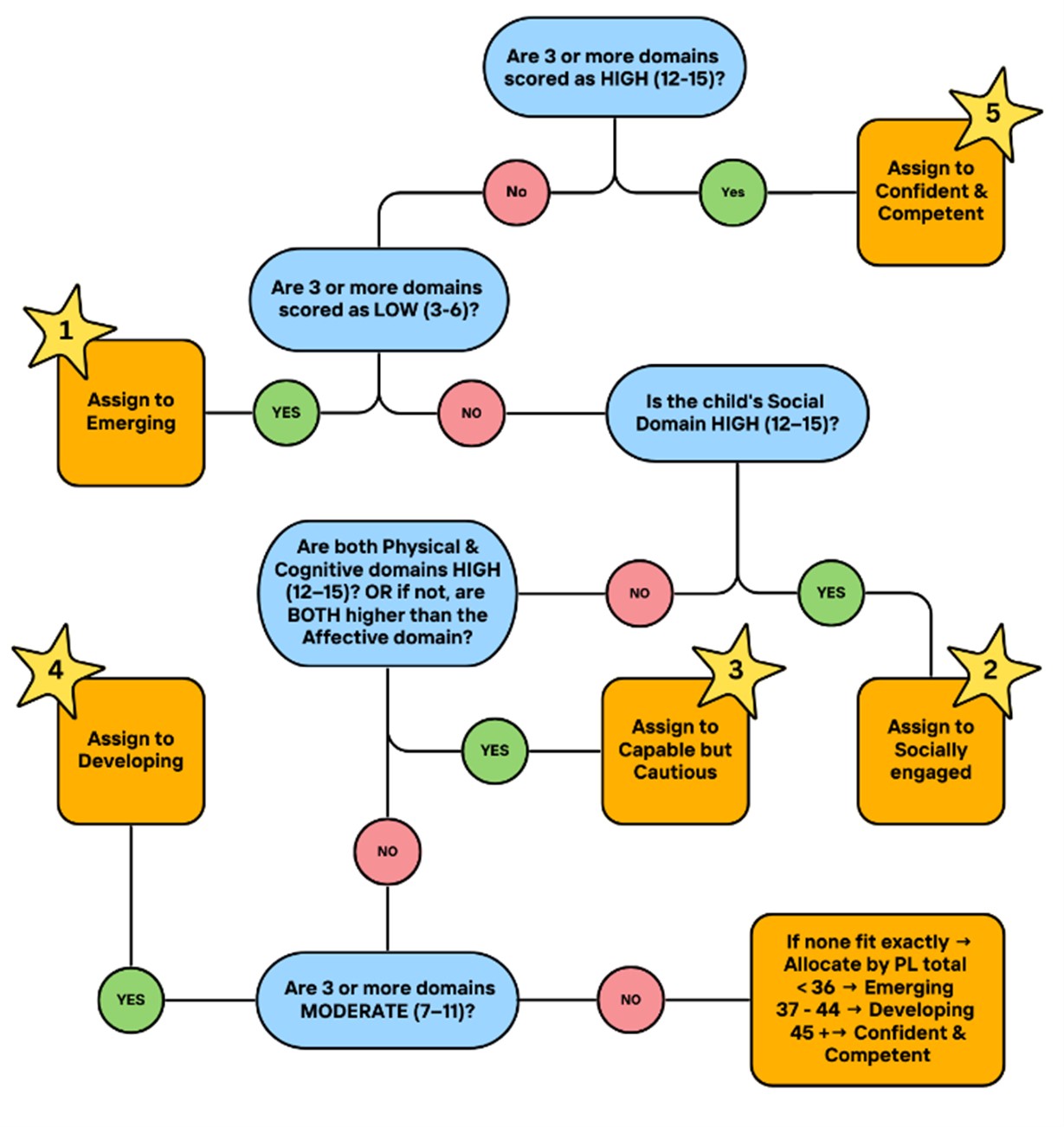

Supplement: Supplementary Material 2 — Physical literacy early years (PLEY) Wheel decision tree. [file Image1.jpeg]
